# Supplementary material for: Unique features of transcription termination and initiation at closely spaced tandem human genes
Source: Mol Syst Biol. 2022 Apr 1;18(4):e10682. doi: 10.15252/msb.202110682 (PMC8972054; doi:10.15252/msb.202110682)
Supplement: Supplementary file 1 — Appendix [file MSB-18-e10682-s001.pdf]

Table of contents

Page
2 Appendix Figure S1. Enriched motifs in STIRs.
4 Appendix Figure S2. Pol2 marks at STIRs sense and antisense strands.
5 Appendix Figure S3. Evidence for elongating Pol2 at STIRs.
6 Appendix Figure S4. KD candidates for the transcription regulation of downstream tandem genes.
8 Appendix Figure S5. Pol2 marks at increasing lengths of tandem intergenic regions.
9 Appendix Figure S6. Cluster analysis of STIR normalized to promoter control.
11 Appendix Figure S7. Cluster analysis of STIR normalized to 3’ control.

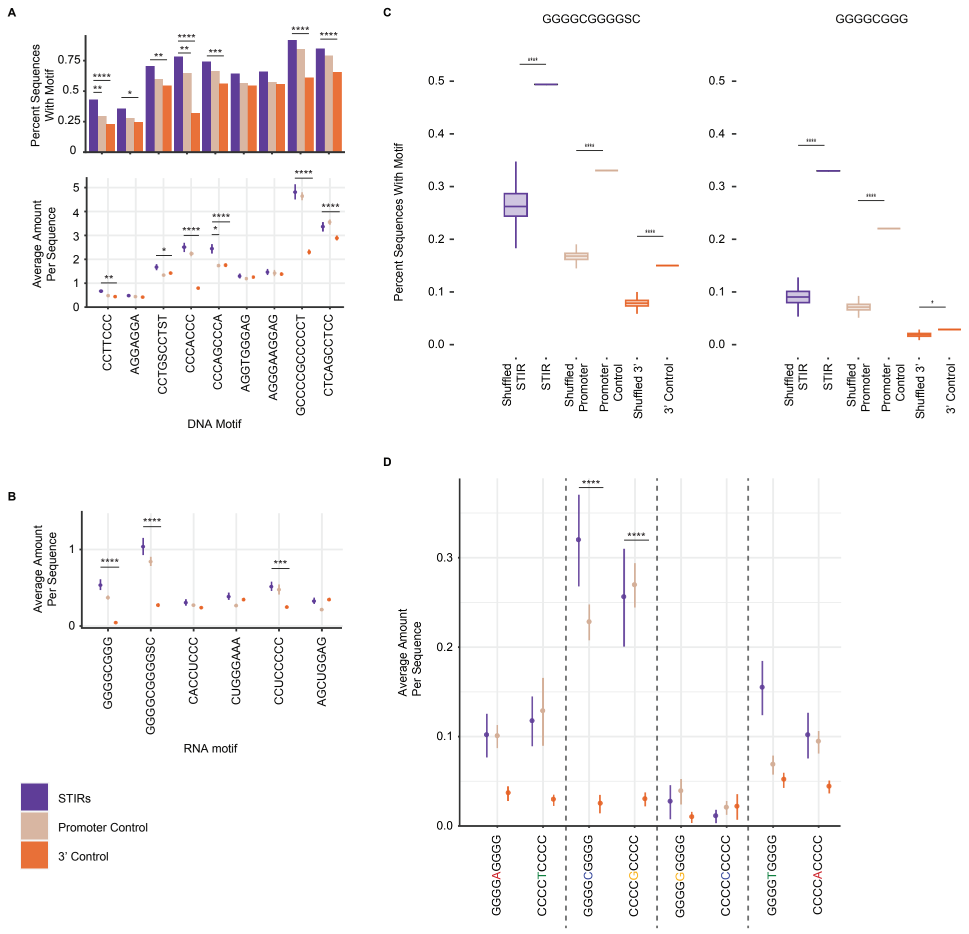

Appendix Figure S1. Enriched motifs in STIRs.

(A) Top: barplots showing the proportion of STIRs (purple) or control sequences (beige and orange) that carry the DNA-mode STREME-discovered motifs in either HepG2 or K562 defined co-expressed tandem genes and their respective mean aggregated controls. Shown are Bonferroni corrected proportion test P-values (*:P<=0.05, **:P<=0.01, ***:P<=0.001, ****:P<=0.0001). Bottom plots show the overall average number and standard error of motif occurrences at STIRs or control sequences. P-values were obtained using paired Wilcoxon rank-sum test and corrected using Bonferroni correction.

(B) As in the bottom plot of (A), but for the RNA-mode STREME sequence motifs (***:P<=0.001, ****:P<=0.0001).

(C) Boxplots showing the fraction of dinucleotide-preserving shuffled sequences of STIRs or controls that contain either of the G-rich motifs (GGGGCGGGGSC motif found based on 164 co-expressed tandem pairs in K562 cell line and GGGGCGGG found in HepG2 based on 188 tandem pairs, each of them compared to their respective controls), based on 1,000 shuffling iterations. Bars correspond to the prevalence of either motif in the actual sequences. The thickened line in the boxplots

represents the median percent of shuffled sequences that carry the respective sequence, the lower and upper boxplot hinges correspond to first and third quartiles of the data, respectively. The whiskers represent the minimal/maximal existing value within 1.5 × inter-quartile range. Outliers were removed from the analysis. Shown also is the calculated Z-test P-value between occurrence prevalence at each group and its shuffled sequences (*:P<=0.05, ****:P<=0.0001).

(D) As in the bottom plot of (A), but for the G_4NG_4 motifs (or their reverse complement) in 188 tandem pairs defined HepG2 cells or their mean aggregated controls. P-values were obtained using paired Wilcoxon rank-sum test and corrected using Bonferroni correction (****:P<=0.0001).

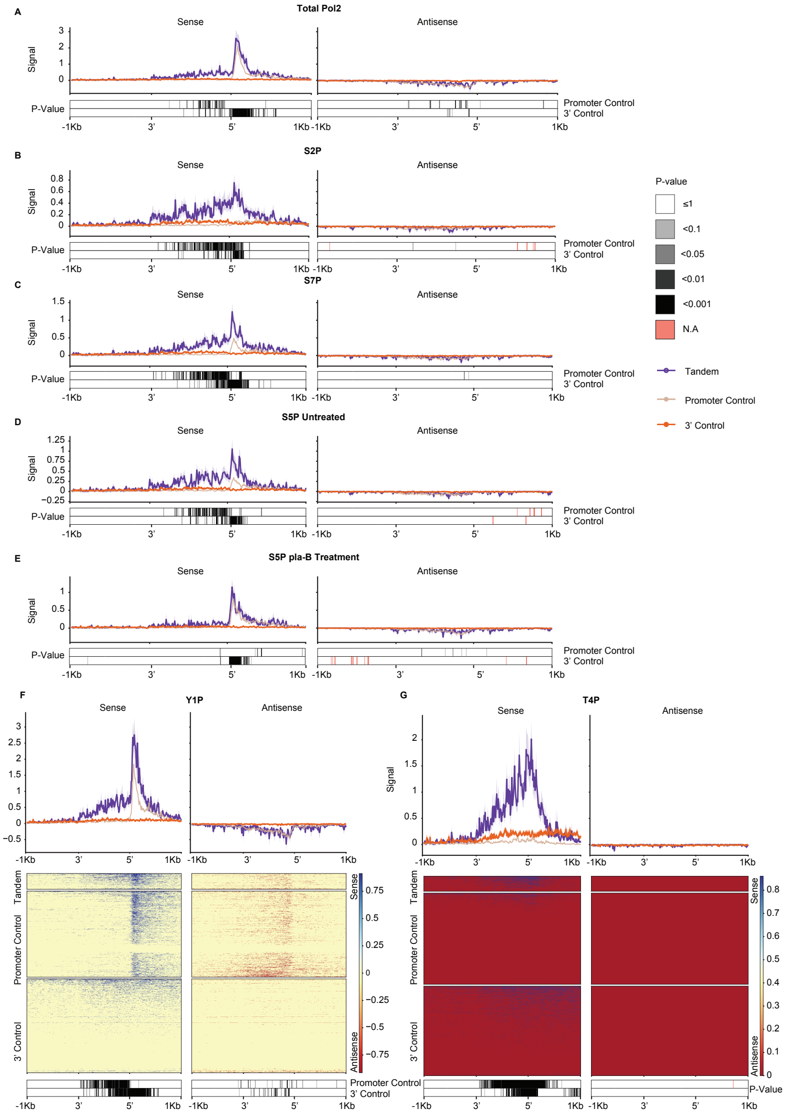

Appendix Figure S2. Pol2 marks at STIRs sense and antisense strands.

(A-E) Metagene analysis (top) showing the different median Pol2 carboxy-terminal domain modifications (phosphorylated and non-phosphorylated, S2P, S7P, S5P and S5P+Pla-B, respectively) occupancy signal and standard error at the sense and antisense strand of STIRs or controls and their flanking regions. Bottom are the respective corrected paired Wilcoxon rank-sum test P-values. NA P-values are marked in cases where data is missing. Data from Schlackow *et al.*, 2017.

(F-G) Metagene analysis (top) and corresponding heatmap (center) of median Pol2 CTD modifications (Y1P and T4P, respectively) mNET-seq signal and standard error at the sense and antisense strands of STIRs and controls, and their flanking regions. Negative values indicate transcription on the antisense strand. Bottom are the respective corrected paired Wilcoxon rank-sum test P-values. Data from Schlackow *et al.*, 2017.

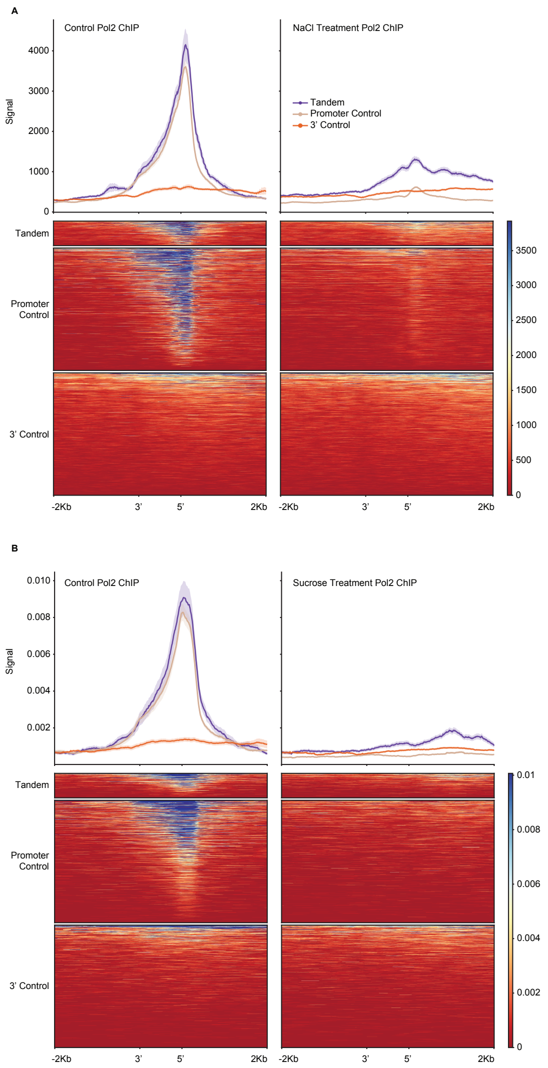

Appendix Figure S3. Evidence for elongating Pol2 at STIRs.
(A) Profile (top) and metagene plot (bottom) for Pol2 ChIP for control treated (left) or NaCl treated (right) HCT116 cells (using Rabbit anti-pan CTD antibody; (Erickson et al. 2018), (Schroeder et al. 2000)). Plotted are the STIRs (purple) or the control promoters (beige) or control CPAs (orange) and flanking 2kb within the gene bodies. (B) As in (A), but for Sucrose treatment.

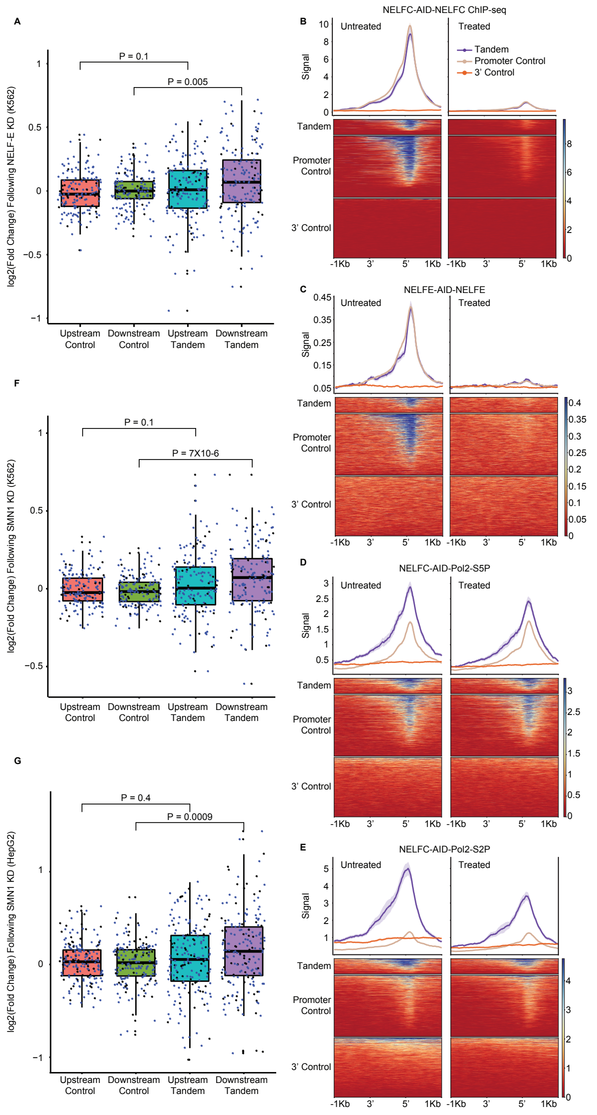

Appendix Figure S4. KD candidates for the transcription regulation of downstream tandem genes.

(A) Boxplot of expression changes in upstream or downstream 164 co-expressed tandem genes following NELF-E KD in K562 cells (Data from ENCODE) or their controls (5 mean aggregated controls per tandem gene). Blue dots correspond to tandem pairs co-expressed in both K562 and HepG2 cell lines (or their respective control). Black dots are tandem genes co-expressed only in the respective cell line. The thickened line represents the median log2 fold change following KD, the lower and upper boxplot hinges correspond to first and third quartiles of the data, respectively. The whiskers represent the minimal/maximal existing value within 1.5 × inter-quartile range. Outliers were removed from the analysis. P-values were obtained using paired Wilcoxon rank-sum tests.

(B) Metagene analysis (top) and corresponding binding heatmap (bottom) of NELFC median ChIP-seq signal for NELFC AID-treated (right), or non-treated (left) data at

STIRs between genes co-expressed in Dld1 cells and flanking 5' and 3' regions and at the control regions.

(C) As in (B), but for NELF-E.

(D-E) As in (B), but ChIP-seq was done for Pol2 S5P (D) and S2P (E) modification following NELF-C KD.

(F) Same as (A), but for SMN1 KD in the K562 cell line.

(G) Same as (F), but for KD experiment in HepG2 cell line (188 tandem genes were considered per group, 5 mean aggregated controls per tandem gene).

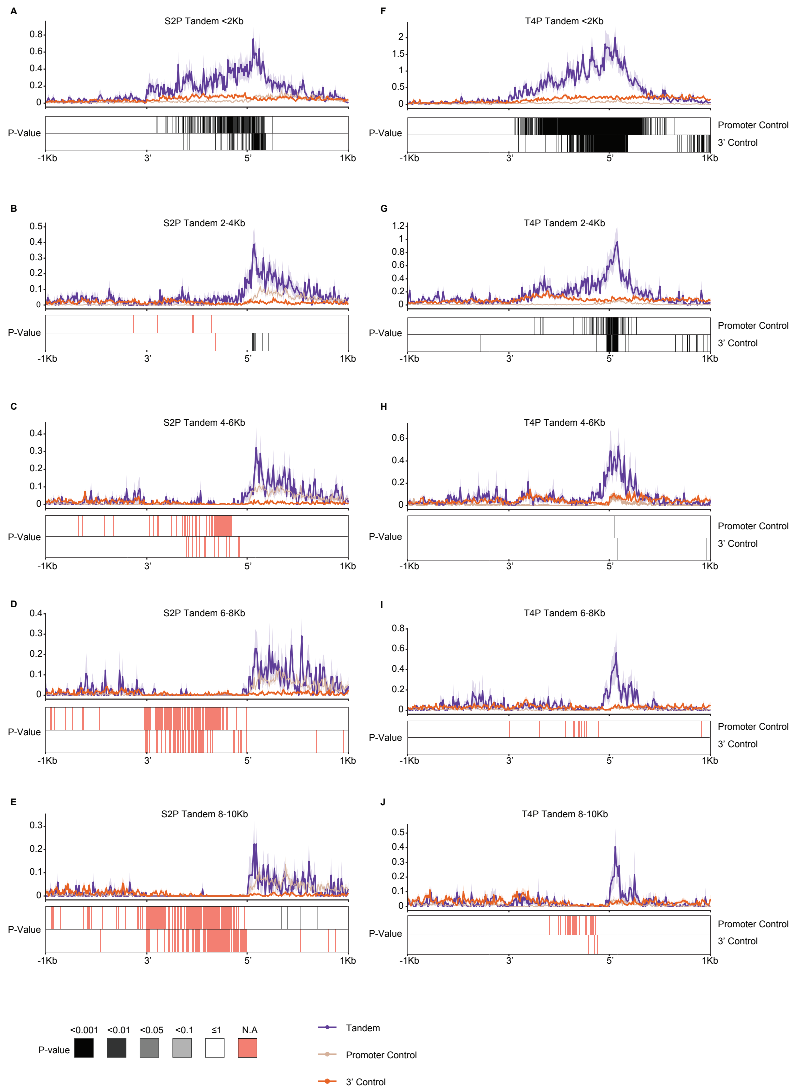

Appendix Figure S5. Pol2 marks at increasing lengths of tandem intergenic regions.

(A-E) Metagene plot showing Pol2 CTD S2P marks at increasing tandem intergenic lengths (<2kb, 2–4kb, 4–6kb, 6–8kb and 8–10kb) and their respective controls (top). Bottom heatmap shows the binned Bonferroni-corrected paired Wilcoxon rank-sum test P-value heatmap (NA is indicated in cases of missing signal).

(F-J) As in (A-E), but for T4P CTD modification.

Maximal Tandem Value Divided By Maximal Value of Promoter Control

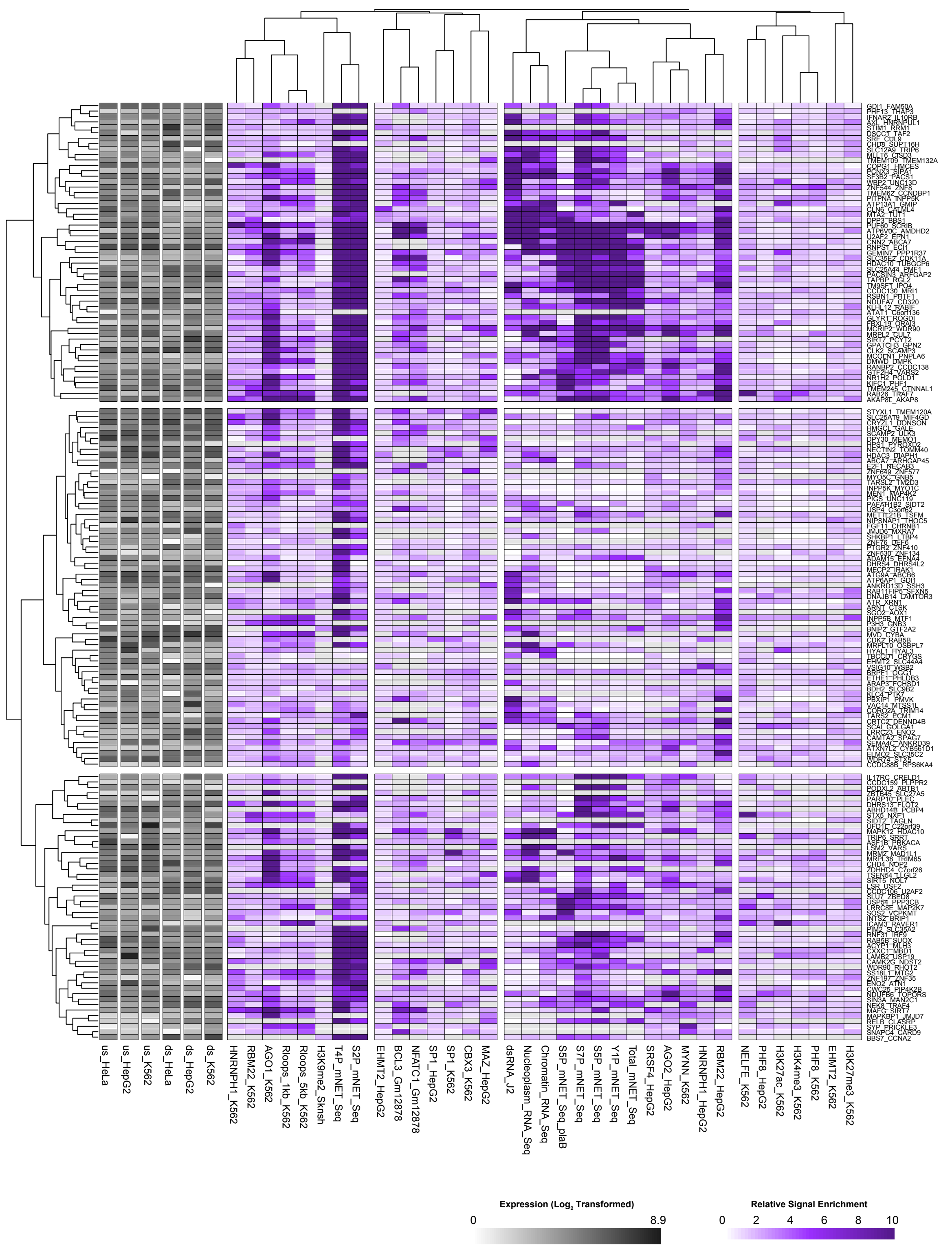

Appendix Figure S6. Cluster analysis of STIR normalized to promoter control. Maximal signal per STIR and flanking regions was calculated for 171 tandem genes or their controls (y-axis) per experiment tested in Figures 2-6, EV1-EV3, and EV5. Matrix of maximal signal at STIRs was divided by the matching matrix of promoter controls. Clustering of the experiments was done using Pearson’s correlation.

Clustering of STIRs was done by computing the Euclidean distance. Left annotations show the log_2-transformed expression levels of the respective genes within the different cell lines.

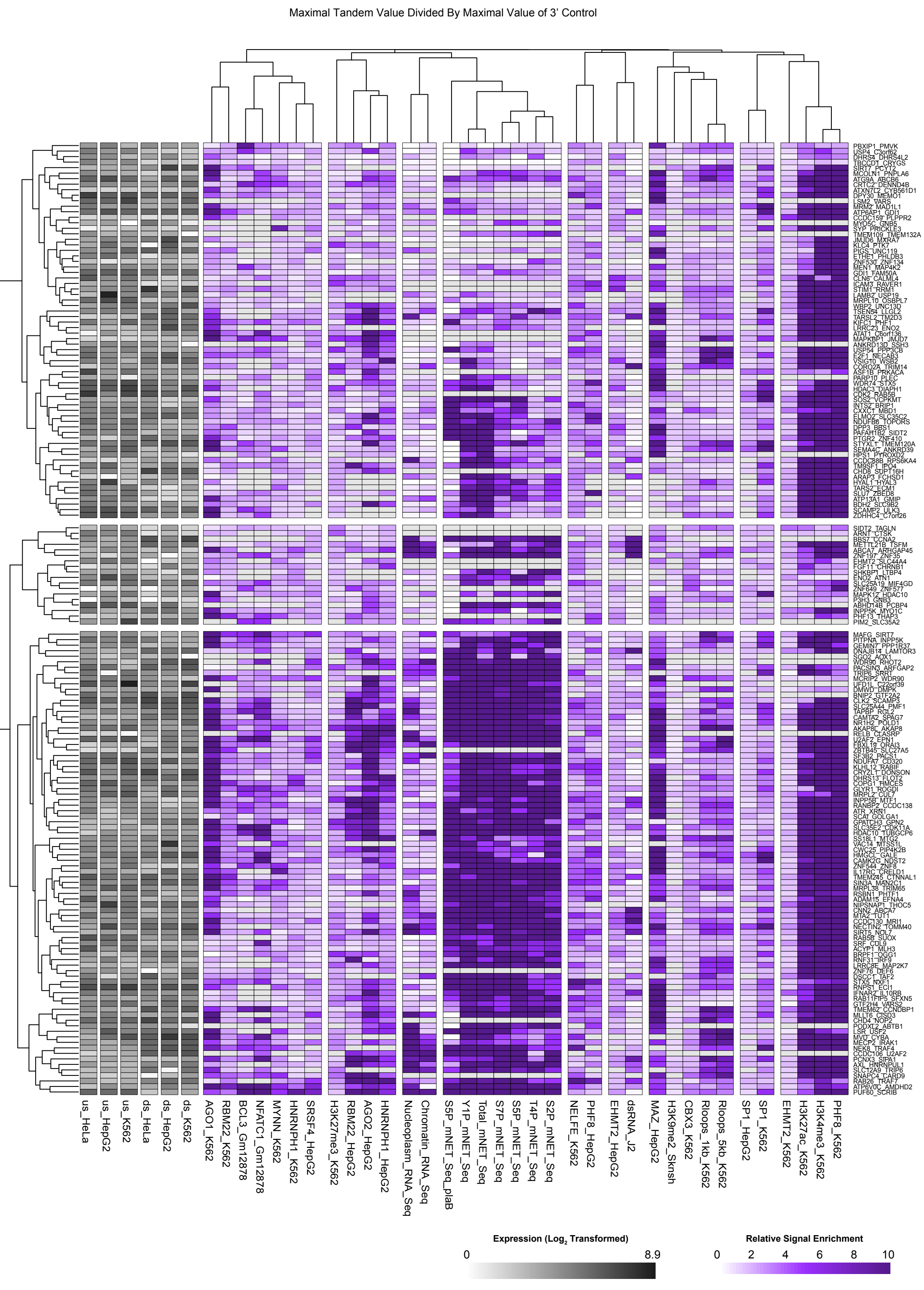

Appendix Figure S7. Cluster analysis of STIR normalized to 3’ control. Maximal signal per STIR and flanking regions was calculated for 171 tandem genes or their controls (y-axis) per experiment tested in Figures 2-6, EV1-EV3, and EV5. Matrix of maximal signals at STIRs was divided by the matching matrix of 3’ controls.

Clustering of the experiments was done using Pearson’s correlation. Clustering of STIRs was done by computing the Euclidean distance. Left annotations show the log_2-transformed expression levels of the respective genes within the different cell lines.
