# Supplementary material for: Unique features of transcription termination and initiation at closely spaced tandem human genes
Source: Mol Syst Biol. 2022 Apr 1;18(4):e10682. doi: 10.15252/msb.202110682 (PMC8972054; doi:10.15252/msb.202110682)
Supplement: Supplementary file 2 — Expanded View Figures PDF [file MSB-18-e10682-s010.pdf]

Expanded View Figures

Figure EV1. Definition of controls.

A Scatter plot showing the correlation between the TPM expression of downstream and upstream tandem gene pairs in HepG2 cell line (based on ENCODE data). Calculated Spearman correlation R = 0.24 with Bonferroni corrected *P*-value of 0.007. Black line is x = y.

B An example of the set of controls for the U2AF2-EPN1 tandem pair separated by 479 nucleotides (top). U2AF2 upstream tandem gene was matched with five 3′ controls having the closest total intron length (averaged over the different RefSeq transcripts) combined with similar expression (center). EPN1 downstream tandem gene was matched with five promoter controls in the same manner (bottom).

C Sense and antisense metagene profile (top) and heatmap (center) of median and standard error chromatin RNA-seq signal based on data from (Schlackow *et al*, 2017). Profile of the STIRs in HeLa cell line is plotted in between the CPA site of the upstream gene (5′) to the TSS of the downstream tandem gene (3′) and in 1 kb flanking sequence. For the control genes, the TSSes of promoter control genes are aligned to the TSSes of the downstream genes (beige), and the CPA sites of 3′ control genes are aligned to the CPA sites of the upstream genes (orange). Negative values indicate transcription on the antisense strand. Bottom plot shows the heatmap of the binned Bonferroni-corrected paired Wilcoxon rank-sum test *P*-values.

D, E Boxplot of splicing efficiency of the whole gene (C), or of the first intron (D) in co-expressed tandem genes or their controls, defined based on HepG2 data (188 genes in each tandem group, 5 averaged controls per tandem gene). The thickened line represents the median measured value, the lower and upper boxplot hinges correspond to first and third quartiles of the data, respectively. The whiskers represent the minimal/maximal existing values within 1.5 × inter-quartile range. Outliers were removed from the analysis. Values were tested using a Bonferroni-corrected paired Wilcoxon rank-sum test (****P* ≤ 0.001).

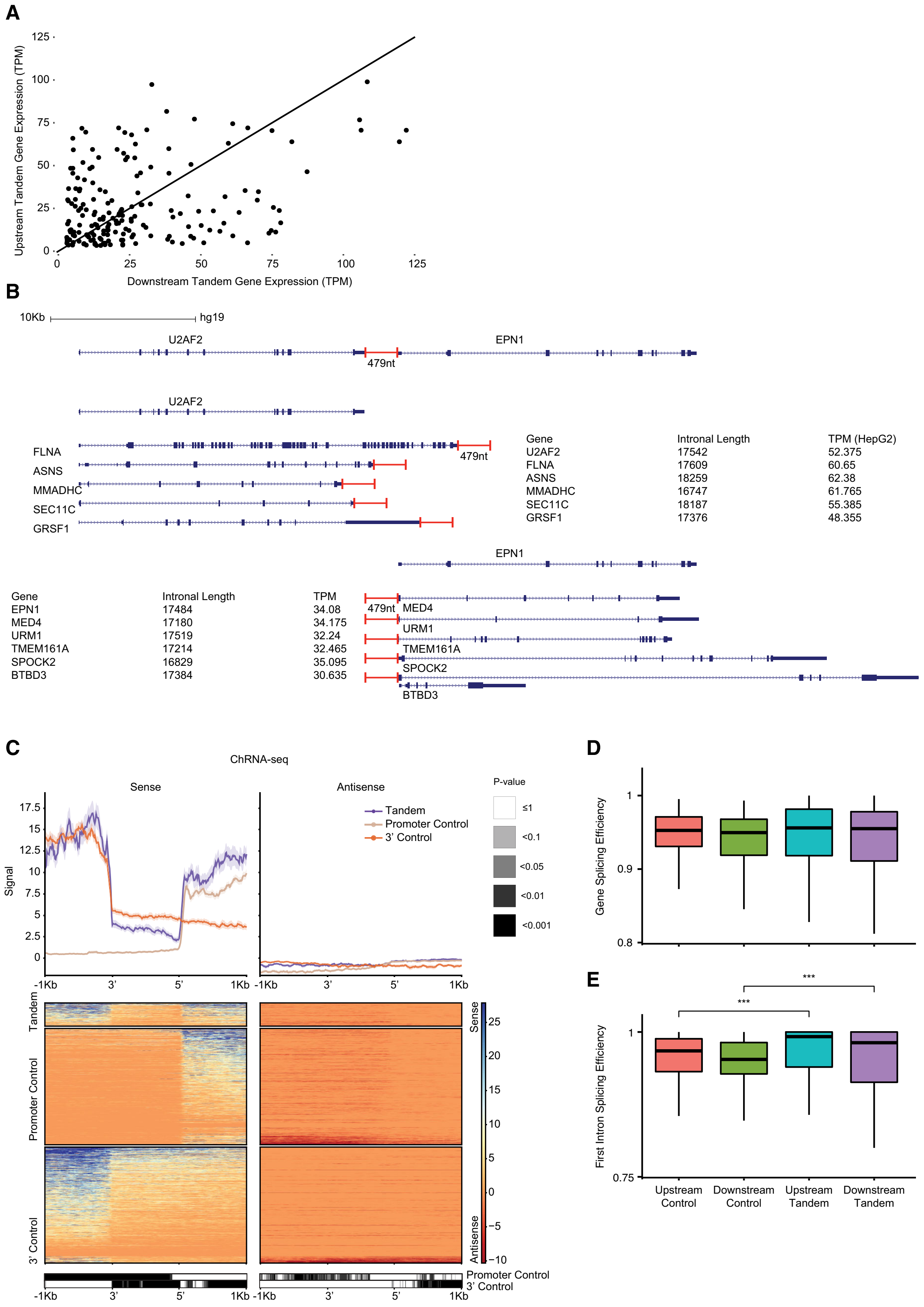

Figure EV1.

 © 2022 The Authors

Figure EV2. Enriched binding of proteins at STIRs.

A, B Scatter plot showing the proportion of ChIP-seq–based binding sites at promoter- (x-axis) or 3′- (y-axis) control sequences in HepG2 (A) or K562 (B) cells. Marked are selected proteins of interest or proteins with relatively high proportions in both axes. Black diagonal line represents equal proportions.

C Barplot showing the proportion of co-expressed STIRs (purple), promoter control- (beige), and 3′ control- (orange) sequences bound by the different proteins (analyzed in K562 cells). Bound sequences are aggregated and counted once in cases multiple binding sites per sequence were suggested. Shown are only proteins with higher binding frequency at STIRs over both controls. Proteins are ordered by STIR ranked frequency and ranked in descending order of calculated minimal ratio of frequencies between STIR and each control. Inset scatter plot shows the log-transformed proportion ratio between binding sites at STIRs or at either controls, in purple are proteins enriched in STIRs over both types of controls.

D Scatter plot showing the number of K562 co-expressed STIRs bound by each protein (as in (C)) versus the minimal tandem-to-control ratio calculated for each control. Indicated are the top enriched proteins at STIRs.

E, F Metagene analysis (top) and corresponding binding heatmap (center) of median ChIP-seq signal of selected STIR-binding protein candidates MYNN (E), and NFATC1 (F). Bottom heatmap shows the corresponding binned paired Wilcoxon rank-sum tests (Bonferroni corrected).

© 2022 The Authors

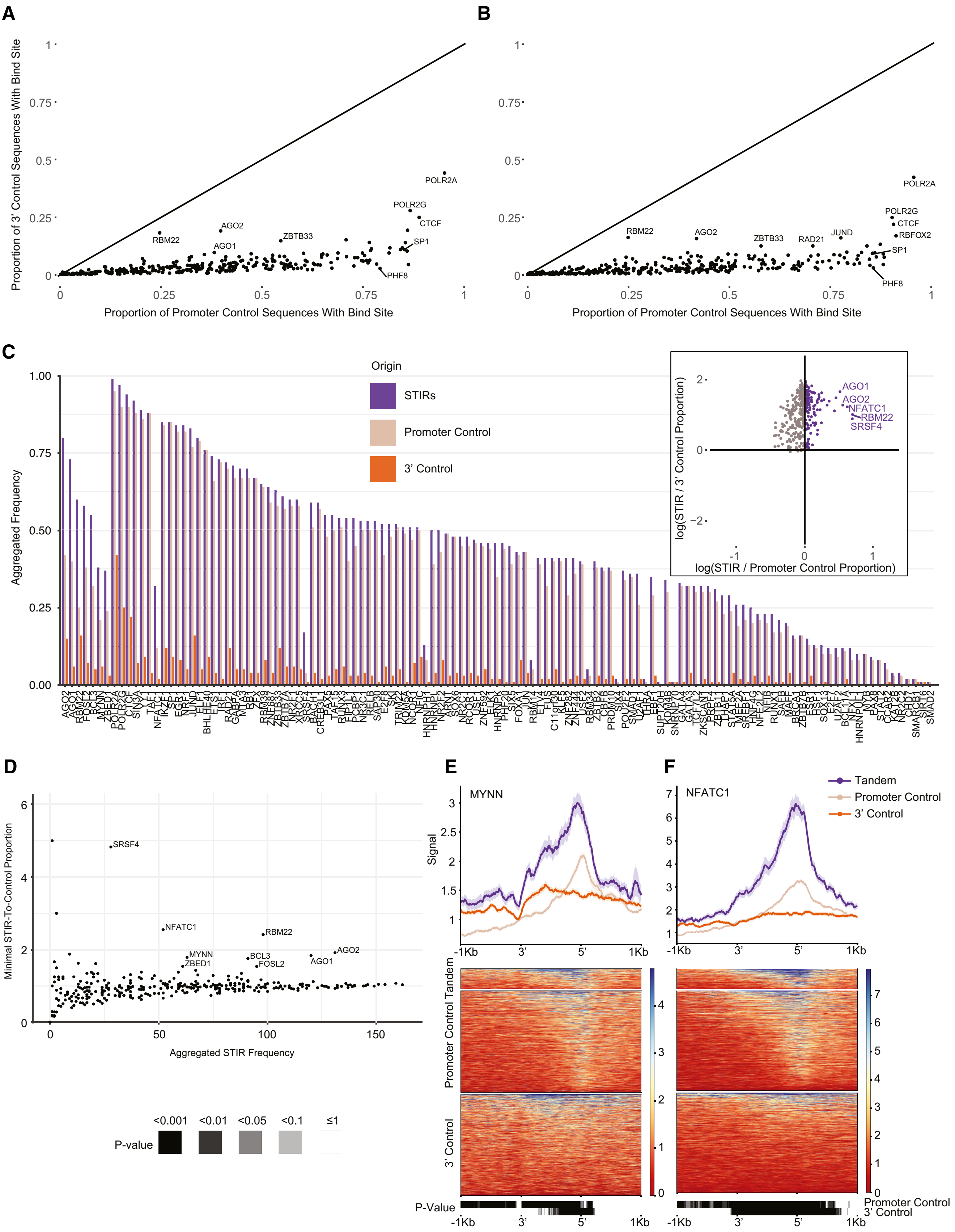

Figure EV2.

 © 2022 The Authors

Figure EV3. Features promoting the proper expression of tandem gene pairs.

A–D Metagene analysis (top) and corresponding binding heatmap (center) of median ChIP-seq signal of the H3K9me2-related factors G9a (A), HP1γ (B), H3K9me2 (C) and PHF8 (D). Bottom plots are the corresponding heatmaps of the binned paired Wilcoxon rank-sum tests (Bonferroni corrected).

E Boxplot of expression changes in upstream or downstream co-expressed tandem genes following AGO1 knockdown (KD) in HepG2 cells (188 genes in each tandem group, 5 averaged controls per tandem gene) (Data from ENCODE). Blue dots correspond to tandem pairs co-expressed in both K562 and HepG2 cell lines (or their respective controls). Black dots are tandem genes co-expressed only in the respective cell line. The thickened line represents the median $\log_2$ fold change following AGO1 KD, the lower and upper boxplot hinges correspond to first and third quartiles of the data, respectively. The whiskers represent the minimal/maximal existing values within 1.5 × inter-quartile range. Outliers were removed from the analysis. *P*-values were obtained using paired Wilcoxon rank-sum test.

F Scatter plot showing the changes in expression following AGO1 KD in K562 cells of each co-expressed tandem pair. Pearson correlation and *P*-value are indicated. Colors indicate median R-loop signal at the STIR. Spearman correlation between the downstream- or upstream- gene expression changes following KD and median AGO1 R-loop signal was tested, with coefficients of −0.278 and −0.117 and $P = 3 \times 10^{-4}$ and $P = 0.13$, respectively.

G Scatter plot showing the changes in expression following AGO1 KD in HepG2 cells of each co-expressed tandem pair. Pearson correlation and *P*-value are indicated.

H Scatter plot showing the changes in expression following AGO2 KD in K562 cells of each co-expressed tandem pair. Pearson correlation and *P*-value are indicated. Colors represent the median R-loops signal at the intergenic region of each tandem pair plotted. Spearman correlation between the downstream- or upstream- gene expression changes following KD and R-loop median signal at STIRs was calculated and was not significant in both cases ($P = 0.75$ and $P = 0.37$, respectively).

© 2022 The Authors

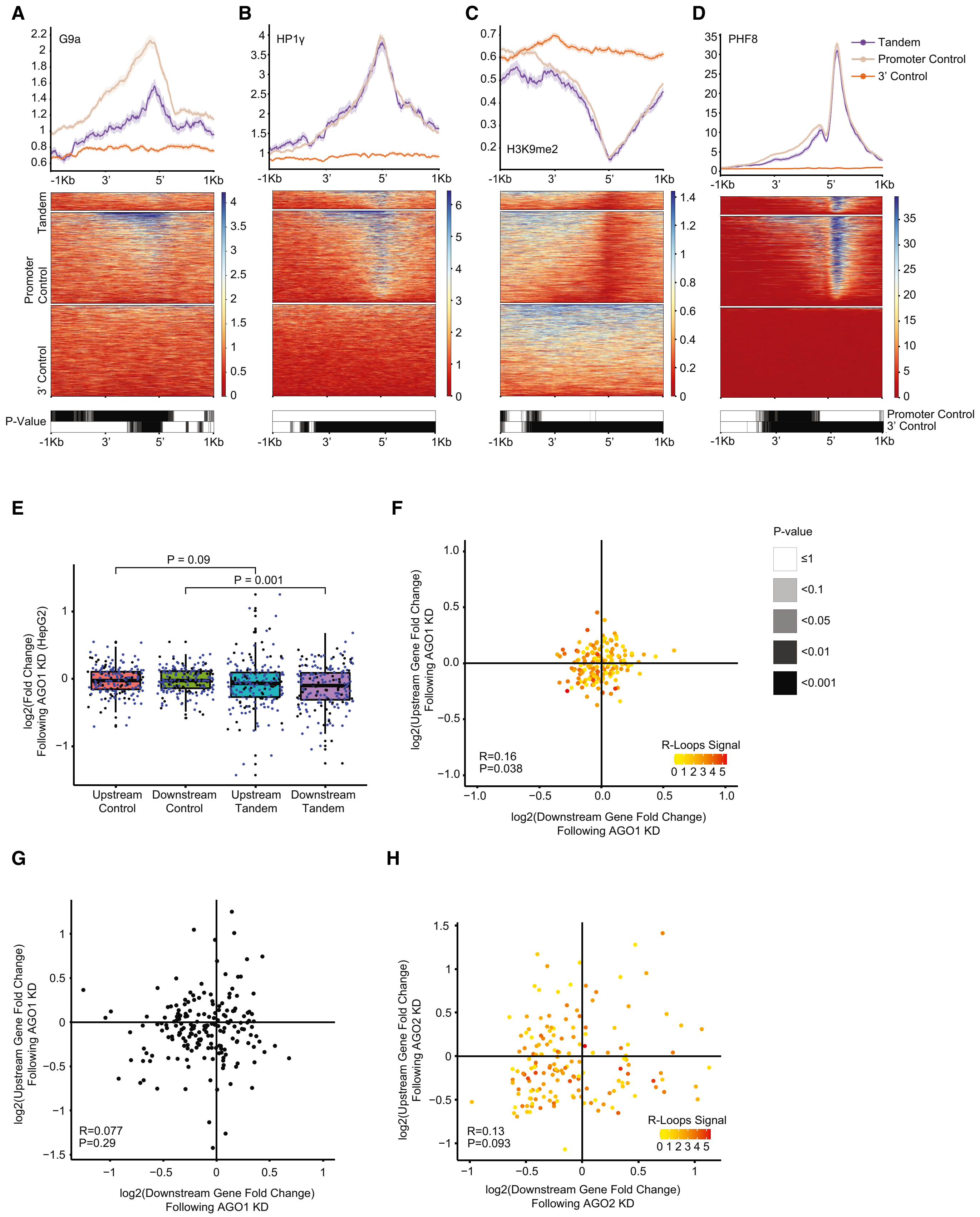

Figure EV3.

© 2022 The Authors

Figure EV4. SPT6 degradation is associated with Pol2 paucity at STIRs.

A Metagene analysis (top) and corresponding heatmap (center) showing mNET-seq median signal following control (DMSO) treatment in K562 co-expressed STIRs and flanking regions and in their controls. Bottom heatmap shows the corrected *P*-value of binned paired Wilcoxon rank-sum test. Data from (Žumer *et al*, 2021), using the CMA601 anti-CTD antibody as in Fig 5 and Appendix Fig S2.

B As in (A), for K562 cells 4 h after treatment with SPT6 dTAG7. Centered heatmap is ordered by the gene order in the heatmap of (A). Right heatmap is the corrected paired Wilcoxon rank-sum test per gene before and after treatment. Proportion of significantly changed signal for genes following the KD treatment was tested between the group of tandem genes and each control group and was significant in both cases ($P = 4.4 \times 10^{-31}$ and $P = 1.1 \times 10^{-9}$ for the promoter or the 3′ control, respectively).

C As in (A), controls for the RTF1 1 h treatment with DMSO.

D As in (C), 1 h following RTF1 dTAG7 treatment. Proportion of significantly changed signal for genes following the KD treatment was tested between the group of tandem genes and each control group and had $P = 0.57$ and $P = 1.6 \times 10^{-4}$ for the promoter or the 3′ control, respectively.

E Boxplots showing the Pol2 pausing index for upstream- (turquoise) or downstream- (purple) tandem genes (164 genes in each tandem group, 5 averaged controls per tandem gene) or for their controls (red and purple, respectively), for either DMSO- (dark), or SPT6 KD- (light) treatment. The thickened line represents the median measured value, the lower and upper boxplot hinges correspond to first and third quartiles of the data, respectively. The whiskers represent the minimal/maximal existing values within 1.5 × inter-quartile range. Outliers were removed from the analysis.

F, G mNET-seq signal over promoter regions (F) or mean signal across whole gene (G) (colors are as in (E)).

Data information: (E–G) Shown are Bonferroni corrected paired Wilcoxon rank-sum test *P*-values (***$P \leq 0.001$, ****$P \leq 0.0001$).

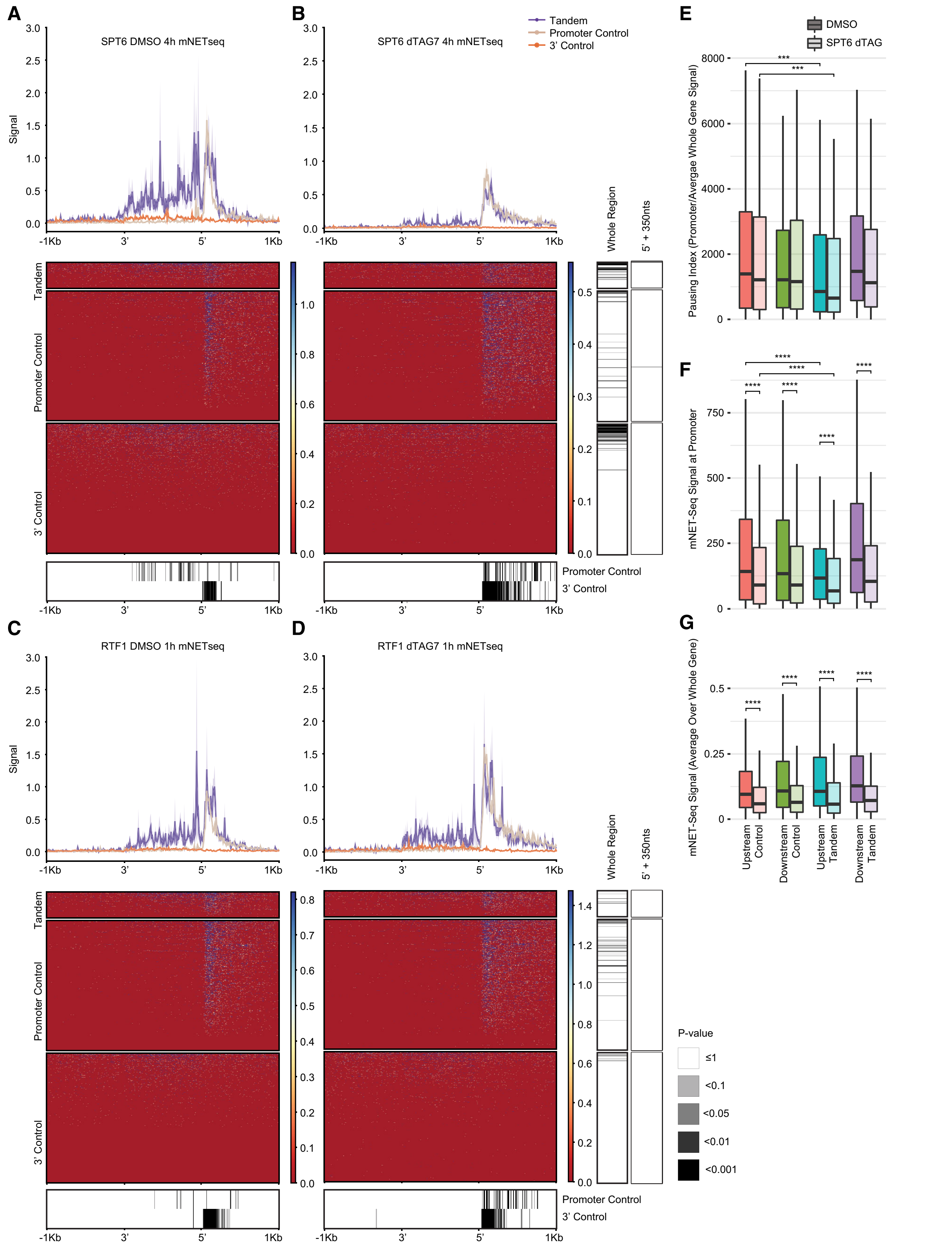

Figure EV4.

© 2022 The Authors

Figure EV5. ChIP-seq signal of histone marks at STIRs.

A–F Metagene plots (top), heatmaps (center) and paired Wilcoxon rank-sum tests Bonferroni-corrected binned *P*-value heatmaps (bottom) of ChIP-Seq signal of different histone modifications H3K27me3 (K562) (A), H3K27ac (K562) (B), H3K9me2 (SK-N-SH) (C), H3K4me3 (K562) (D), H3K36me3 (K562) (E) and H3K79me2 (HepG2) (F). Data from ENCODE.

© 2022 The Authors

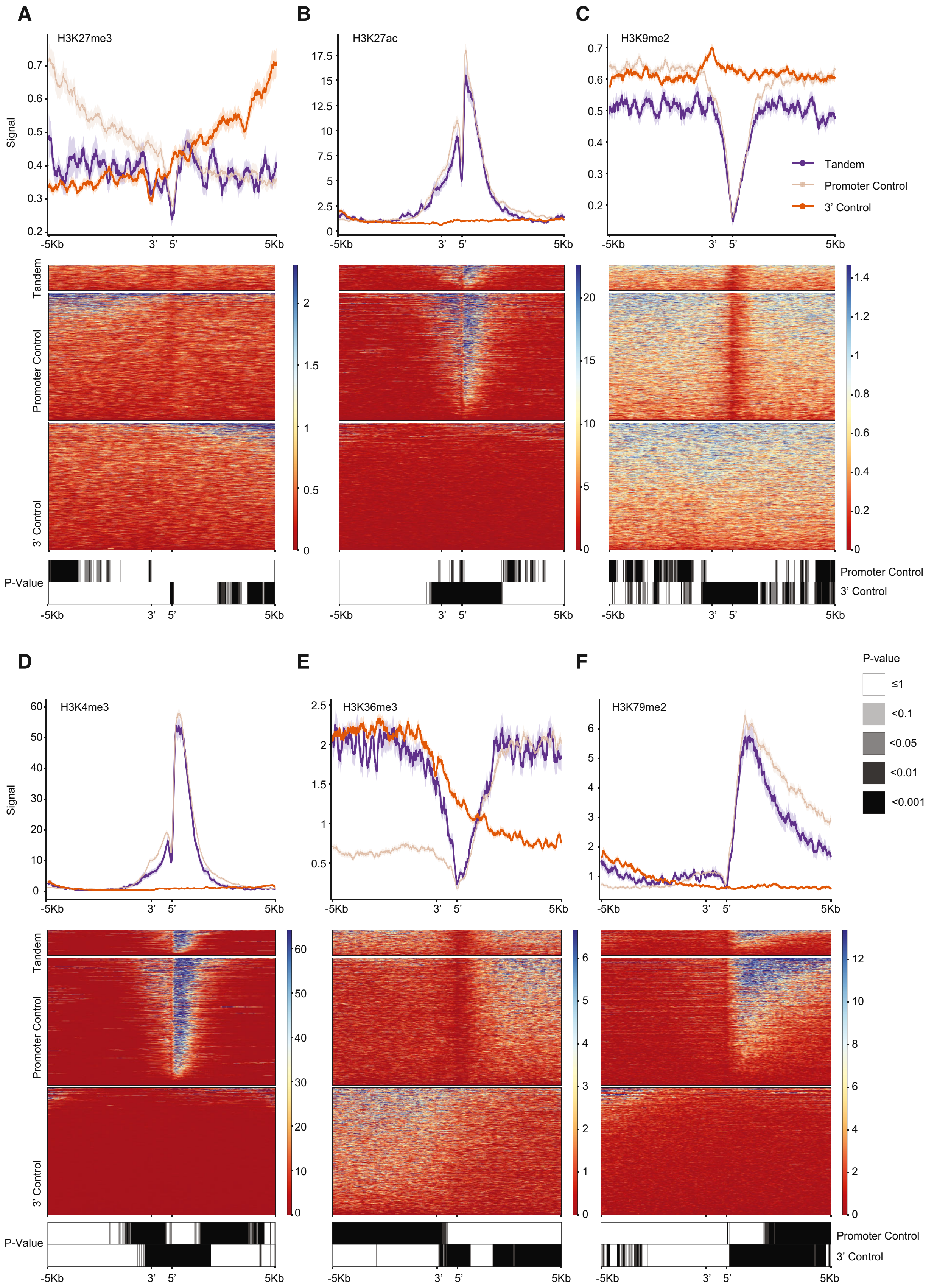

Figure EV5.

© 2022 The Authors
