# Supplementary material for: Unique features of transcription termination and initiation at closely spaced tandem human genes
Source: Mol Syst Biol. 2022 Apr 1;18(4):e10682. doi: 10.15252/msb.202110682 (PMC8972054; doi:10.15252/msb.202110682)
Supplement: Supplementary file 18 [file MSB-18-e10682-s005.pdf]

molecular systems biology

Unique features of transcription termination and initiation at closely spaced tandem human genes

Noa Nissani and Igor Ulitsky
DOI: 10.15252/msb.202110682

Corresponding author(s): Igor Ulitsky (igor.ulitsky@weizmann.ac.il)

Review Timeline:

Submission Date:	9th Sep 21
Editorial Decision:	18th Oct 21
Revision Received:	3rd Feb 22
Editorial Decision:	2nd Mar 22
Revision Received:	9th Mar 22
Accepted:	10th Mar 22

Editor: Jingyi Hou

Transaction Report:

(Note: With the exception of the correction of typographical or spelling errors that could be a source of ambiguity, letters and reports are not edited. Depending on transfer agreements, referee reports obtained elsewhere may or may not be included in this compilation. Referee reports are anonymous unless the Referee chooses to sign their reports.)

18th Oct 2021

Manuscript Number: MSB-2021-10682
Title: Unique features of transcription termination and initiation at closely spaced tandem human genes
Author: Noa Nissani
Igor Ulitsky

Dear Prof Ulitsky,

Thank you for submitting your work to Molecular Systems Biology. We have now heard back from the three reviewers who agreed to evaluate your study. As you will see below, the reviewers find the topic of your study of interest. However, they raise substantial concerns about your work, which should be convincingly addressed in a major revision of the present manuscript.

Without reiterating all the points listed below, the most fundamental issues that need to be convincingly addressed are the following:

- The reviewers' concerns about the data analyses should be carefully addressed or clarified.
- Both Reviewers #2 and #3 raised concerns about the conclusion on Pol II recycling. We would strongly encourage you to provide some level of experimental support and/or additional computational analyses to strengthen this conclusion. Further, Reviewer #3 mentioned that the current arguments supporting this conclusion were somewhat difficult to understand, which needs to be addressed.

All other issues raised by the reviewers need to be satisfactorily addressed as well. As you may already know, our editorial policy allows in principle a single round of major revision and it is therefore essential to provide responses to the reviewers' comments that are as complete as possible.

On a more editorial level, we would ask you to address the following issues:

- Please provide a .docx formatted version of the manuscript text (including legends for main figures, EV figures and tables). Please make sure that the changes are highlighted to be clearly visible.

- Please provide individual production quality figure files as .eps, .tif, .jpg (one file per figure).

-Please provide a .docx formatted letter INCLUDING the reviewers' reports and your detailed point-by-point responses to their comments. As part of the EMBO Press transparent editorial process, the point-by-point response is part of the Review Process File (RPF), which will be published alongside your paper.

-Please note that all corresponding authors are required to supply an ORCID ID for their name upon submission of a revised manuscript.

-We replaced Supplementary Information with Expanded View (EV) Figures and Tables that are collapsible/expandable online (see examples in http://msb.embopress.org/content/11/6/812). A maximum of 5 EV Figures can be typeset. EV Figures should be cited as 'Figure EV1, Figure EV2" etc... in the text and their respective legends should be included in the main text after the legends of regular figures.

Additional Tables/Datasets should be labeled and referred to as Table EV1, Dataset EV1, etc. Legends have to be provided in a separate tab in case of .xls files. Alternatively, the legend can be supplied as a separate text file (README) and zipped together with the Table/Dataset file.

For the figures and tables that you do NOT wish to display as Expanded View figures, they should be bundled together with their legends in a single PDF file called *Appendix*, which should start with a short Table of Content. Each legend should be below the corresponding Figure/Table in the Appendix. Appendix figures and tables should be referred to in the main text as: "Appendix Figure S1, Appendix Figure S2, Appendix Table S1" etc. See detailed instructions regarding expanded view here: https://www.embopress.org/page/journal/17444292/authorguide#expandedview.

-Before submitting your revision, primary datasets (and computer code, where appropriate) produced in this study need to be deposited in an appropriate public database (see https://www.embopress.org/page/journal/17444292/authorguide#dataavailability).

Please remember to provide a reviewer password if the datasets are not yet public.

The accession numbers and database should be listed in a formal "Data Availability" section (placed after Materials & Method) that follows the model below (see also https://www.embopress.org/page/journal/17444292/authorguide#dataavailability). Please note that the Data Availability Section is restricted to new primary data that are part of this study.

Data availability

The datasets (and computer code) produced in this study are available in the following databases:

- RNA-Seq data: Gene Expression Omnibus GSE46843 (https://www.ncbi.nlm.nih.gov/geo/query/acc.cgi?acc=GSE46843)
- [data type]: [name of the resource] [accession number/identifier/doi] ([URL or identifiers.org/DATABASE:ACCESSION])

*** Note - All links should resolve to a page where the data can be accessed. ***

- We would encourage you to include the source data for figure panels that show essential quantitative information. Additional information on source data and instruction on how to label the files are available at <
https://www.embopress.org/page/journal/17444292/authorguide#sourcedata
>.

- All Materials and Methods need to be described in the main text. We would encourage you to use 'Structured Methods', our new Materials and Methods format. According to this format, the Material and Methods section should include a Reagents and Tools Table (listing key reagents, experimental models, software and relevant equipment and including their sources and relevant identifiers) followed by a Methods and Protocols section in which we encourage the authors to describe their methods using a step-by-step protocol format with bullet points, to facilitate the adoption of the methodologies across labs. More information on how to adhere to this format as well as downloadable templates (.doc or .xls) for the Reagents and Tools Table can be found in our author guidelines: < https://www.embopress.org/page/journal/17444292/authorguide#researcharticleguide>. An example of a Method paper with Structured Methods can be found here: .

-Regarding data quantification:
Please ensure to specify the name of the statistical test used to generate error bars and P values, the number (n) of independent experiments (please specify technical or biological replicates) underlying each data point and the test used to calculate p-values in each figure legend. Discussion of statistical methodology can be reported in the materials and methods section, but figure legends should contain a basic description of n, P and the test applied.
Graphs must include a description of the bars and the error bars (s.d., s.e.m.).
Please also include scale bars in all microscopy images.

- Please provide a "standfirst text" summarizing the study in one or two sentences (approximately 250 characters, including space), three to four "bullet points" highlighting the main findings and a "synopsis image" (550px width and 400-600px height, PNG format) to highlight the paper on our homepage.
Here are a couple of examples:
https://www.embopress.org/doi/10.15252/msb.20199356
https://www.embopress.org/doi/10.15252/msb.20209475
https://www.embopress.org/doi/10.15252/msb.209495

When you resubmit your manuscript, please download our CHECKLIST (http://bit.ly/EMBOPressAuthorChecklist) and include the completed form in your submission.
Please note that the Author Checklist will be published alongside the paper as part of the transparent process (https://www.embopress.org/page/journal/17444292/authorguide#transparentprocess).

If you feel you can satisfactorily deal with these points and those listed by the referees, you may wish to submit a revised version of your manuscript. Please attach a covering letter giving details of the way in which you have handled each of the points raised by the referees. A revised manuscript will be once again subject to review and you probably understand that we can give you no guarantee at this stage that the eventual outcome will be favorable.

I look forward to receiving your revised manuscript soon.

Kind regards,
Jingyi

Jingyi Hou
Editor
Molecular Systems Biology

If you do choose to resubmit, please click on the link below to submit the revision online *within 90 days*.

https://msb.msubmit.net/cgi-bin/main.plex

IMPORTANT: When you send your revision, we will require the following items:
1. the manuscript text in LaTeX, RTF or MS Word format
2. a letter with a detailed description of the changes made in response to the referees. Please specify clearly the exact places in the text (pages and paragraphs) where each change has been made in response to each specific comment given
3. three to four 'bullet points' highlighting the main findings of your study
4. a short 'blurb' text summarizing in two sentences the study (max. 250 characters)
5. a 'thumbnail image' (550px width and max 400px height, Illustrator, PowerPoint or jpeg format), which can be used as 'visual title' for the synopsis section of your paper.
6. Please include an author contributions statement after the Acknowledgements section (see https://www.embopress.org/page/journal/17444292/authorguide)
7. Please complete the CHECKLIST available at (https://bit.ly/EMBOPressAuthorChecklist).
Please note that the Author Checklist will be published alongside the paper as part of the transparent process (https://www.embopress.org/page/journal/17444292/authorguide#transparentprocess).
8. Please note that corresponding authors are required to supply an ORCID ID for their name upon submission of a revised manuscript (EMBO Press signed a joint statement to encourage ORCID adoption). (https://www.embopress.org/page/journal/17444292/authorguide#editorialprocess)
9. When assembling figures, please refer to our figure preparation guideline in order to ensure proper formatting and readability in print as well as on screen:
https://bit.ly/EMBOPressFigurePreparationGuideline
See also figure legend guidelines: https://www.embopress.org/page/journal/17444292/authorguide#figureformat

Currently, our records indicate that the ORCID for your account is 0000-0003-0555-6561.

Please click the link below to modify this ORCID:
Link Not Available

The system will prompt you to fill in your funding and payment information. This will allow Wiley to send you a quote for the article processing charge (APC) in case of acceptance. This quote takes into account any reduction or fee waivers that you may be eligible for. Authors do not need to pay any fees before their manuscript is accepted and transferred to the publisher.

As a matter of course, please make sure that you have correctly followed the instructions for authors as given on the submission website.

*** PLEASE NOTE *** As part of the EMBO Press transparent editorial process initiative (see our Editorial at https://dx.doi.org/10.1038/msb.2010.72), Molecular Systems Biology publishes online a Review Process File with each accepted manuscripts. This file will be published in conjunction with your paper and will include the anonymous referee reports, your point-by-point response and all pertinent correspondence relating to the manuscript. If you do NOT want this File to be published, please inform the editorial office at msb@embo.org within 14 days upon receipt of the present letter.

Reviewer #1:

In the present study, Nissani & Ulitsky addresses the interesting question of how transcription termination of a gene can impact transcription of a closely spaced downstream gene in human cells. Specifically, the authors investigate whether intergenic regions between closely spaced and co-expressed tandem genes (distance less than 2 kb) possess specific features. To address these questions Nissani & Ulitsky re-analyzed available genome-wide ENCODE data (RNA-seq, ChIP-seq) and mNET-seq data. In a first step, the authors systematically identified gene pairs and found that co-expression was most common in closely spaced (<2 kb) gene pairs. The authors then focused primarily on the 188 closely spaced and co-expressed tandem genes that they identified in human HepG2 cells. Next, the authors investigated if the short intergenic regions between close tandem genes, called STIRs, are characterized by specific features. Notably, this analysis indeed revealed distinct characteristics including sequence features. The authors provide strong evidence for an enrichment of G nucleotides in STIRs and uncovered G-rich sequence motifs. Furthermore, the authors show that these regions are characterized by distinct protein binding events, such as of AGO1 and AGO2, and also provide some evidence that RNA polymerase II has a specific CTD phosphorylation pattern at STIRs.

The computational analyses were carefully performed and the obtained results of this work are exciting. A clear strength of this work is the identification of specific features of STIRs in human cells. The most exciting finding at least for me is the discovery of sequence features including G-rich motifs that are enriched in STIRs. Overall the manuscript is excellently written and main conclusions are supported by the data that is provided. Furthermore, the authors did an exceptional job in creating high-quality figures that make it easy for readers to immediately grasp key findings. This study also opens up interesting future research directions such as to further functionally and mechanistically characterize the sequence elements that the authors uncovered in this study. I have no doubts that the findings of this work will be of broad interest for researchers in the transcription field and to researchers of other disciplines who have an interest in transcription regulation.

Major comments:
(1) The authors analysed RNA-seq data upon knockdown of 245 different factors and focused on factor depletions upon which the expression of the up- or downstream genes were differently affected. The authors then almost exclusively focused on the observations obtained upon NELF-E/NELF complex knockdown. Although, I agree that this complex is of particular interest in this context, the list of factors that is provided in Table S10 also contains other interesting factors that could be mentioned and discussed in the main text.
Please clearly state in the main text how the up- and downstream genes are affected upon NELF-E knockdown. Please also simplify Table S10, if possible, so that the reader immediately recognizes how the expression of the up- and downstream genes are altered upon the respective factor knockdown.

(2) The authors propose a model according to which the STIR region may represents a 'preparation area' for a new transcription cycle of the downstream gene, leading to a recycling of the Pol II that has transcribed the upstream gene. As presented, this model is primarily based on the Pol II T4P occupancy data. If both transcription cycles are coupled, I would have expected that rapid depletion of the key Pol II transcription factor SPT6 not only impacts Pol II density at the STIR region, but also as a consequence in the promoter-proximal region of the downstream gene. However, this seems not to be the case. Although rapid SPT6 depletion induces a collapse of Pol II over the STIR region, the Pol II density in the promoter-proximal region of the downstream gene seems not to be affected (Figure S6A, B). Other potential limitations that need to be considered in this context are: (1) it is likely that Pol II and its CTD isoforms which are detected in the narrow STIR region are a mixed population of terminating (upstream gene) and promoter-bound/initiating Pol II (downstream gene). (2) This study relies on bulk data which provides an average view on Pol II transcription. It remains unclear if the upstream and downstream genes are indeed expressed at the same time in the same cell. This similarly affects the interpretation of the Pol II occupancy data. It would be helpful to clarify this a bit more when presenting the model.

Minor comments:
(1) On page 2 (Introduction) the authors describe the two models of transcription termination: the allosteric and the torpedo model. Please add the corresponding references.

(2) On Page 3 (Introduction) the authors mention that Pol II CTD S2P and T4P have been linked to transcription termination. The authors should mention that Pol II CTD Y1P has also been linked to transcription termination in yeast (PMID: 22745433) and mammals (PMID: 29304333).

(3) Figure 1A: please mention the data source in the figure legend.

(4) Figure 1G: can you please clarify in the figure legend if "3' Upstream gene" means cleavage and polyadenylation site (CAS).

(5) The authors identified G-rich sequence motifs in the short intergenic region (Figure 2A). Do these motifs have any similarity to known transcription factor binding sites (except from MAZ and Sp1)?

(6) Please specify in the text if the antibody that was used to obtain total Pol II mNET-seq (CMA601) is directed/or not directed against the Pol II CTD (to avoid any CTD bias).

(7) Figure 5: please mention the number of replicates in the figure legend.

Reviewer #2:

The authors present an extensive analysis of genomics data focused a few hundred pairs of closely spaced human genes oriented in tandem. They identify several genomic features enriched in these genomics intervals. Some perturbations of transcription correlate with preferential effects on the expression of the downstream gene. The authors conclude that features associated with potential Pol2 recycling may the predominant driving force shaping these regions.

The quality of data analyses is very good. The writing is clear and easy to follow. The figures are well-crafted for the most part help to highlight the key finding. In these respects the manuscript is impressive since it presents an analysis of a large data volume in a coherent style. A satisfactory systems-level understanding of gene regulation in the tandem configuration was arguably not reached. The manuscripts offers no original laboratory experiments to test any of the correlations, which likely limits

the scope, novelty and impact of this manuscript. The conclusions on Pol2 recycling and transcriptional interference are insufficiently supported. On the other hand, many relevant genomics resources have been exhausted to address tandem gene regulation, and so the manuscript may be of interest to genomicists. Comments to improve the manuscript are listed below.

- The authors identified closely spaced tandem genes. In general, the presence of un-annotated and cryptic transcripts (lncRNAs, or transcripts only in nascent Pol2 data) is a potential confounding factor for the interpretation of various signals and signatures. Perhaps I missed it, but I did not find a paragraph in the text and method how this confounding factor was handled. Additional transcription units in SIRTs would have the effect that the selected tandem transcription units may in fact not be tandem, since un-annotated transcripts may be present in the areas. Likewise, others genes that were excluded may in fact be tandem with a cryptic non-coding transcript. Performing the segmentation properly is a complicated task, but it may particularly straightforward to address if the downstream promoters give rise to divergent lncRNA transcription using various resources for nuclear exosome RNA decay factor perturbations, for example (PMID: 32710631, 27455346, 32075771). If so, the upstream transcripts would actually be tail-to-tail with a divergent lncRNA. Essentially, the classification of tandem genes may be confounded by other transcripts that would result in a different segmentation if tandem transcripts (coding and non-coding) were considered.
- Predominantly in early parts of the manuscript a focus appears to be on transcriptional interference. Since the mechanism of transcriptional interference is not restricted to mRNA-mRNA pairs, this segmentation is too crude to justify statements on this mechanism. Either the writing needs to reflect this, or a higher quality segmentation needs to be presented that relies on a fully updated genome annotation including short and long ncRNA transcription units in these regions that may be transcribed on both strands. Data-driven genome annotation in other systems may serve as inspiration here (PMID: 34058980).
- Conclusion Page 4 bottom. It may indicate a logical flaw to expect that transcriptional interference poses a substantial obstruction to transcription from the downstream promoters. In fact, the downstream gene is often expressed at high levels, yet no functional initiation occurs (PMID: 6329902, 15175754, 30707695 and many others). The method to measure expression, it appears to be RNA-seq, should be explicitly stated in the text. In any case, unless the authors rely on appropriate methods here that resolve Pol2 initiation in these regions and have information on the nature of the overlapping transcripts, it is inadequate to make statements about the mechanism of transcriptional interference.
- Page 7, end 1st paragraph and 1G legend. How were the TTS positions determined? Is that information based on mNET-seq data or are the authors referring to poly-(A) positions here? The poly-(A) position is not the equivalent to the TTS. Similar in S1, since most standard genome annotation lack the important read-through tail that could give the range of TTSs, it looks like the red lines are probably p(A) - TSS distances. Using TTS-TSS distances would be much more appropriate, at least for an investigation of transcriptional interference. Perhaps an analyses of POINT or nano-COP (PMID: 33735606, 31839405) may offers some insight into where nascent transcription signal in SIRTs represents terminating transcription from the upstream genes, un-annotated nascent transcripts or cryptic initiation of the downstream genes.
- P.20. Related point, "During transcription termination, the current model is that transcription continues for a few hundreds to several kbs post the TTS before the polymerase is released from the DNA ... " there is some confusion. TTS needs to be replaced with cleavage site where the p(A)-tail is added in this sentence. Transcription continues beyond this position, so it is by definition not terminated yet. The TTS is the termination site, which is often missing in genome annotations but at least a read-through tail that informs on TTSs is annotated for some genomes (PMID: 34058980).
- ChIP-seq data representations in Fig2EF, 3D-G, 4A. The genomic regions used as controls need to be explicitly defined. Presumably these correspond to the same groups as for the intron analyses on top of p.7, but it is not defined in the legends. Also, what justifies the choice of 5x more control regions than target STIRs? Selecting the same n would be more intuitive to allow for equal noise effects in the control regions.
- The factors in Fig2EF, 3D-G, 4A, S3D, S4A,B,D appear to ChIP to the 5´-gene. It would be good to show that some factors specifically enrich for the 3´-end as control, otherwise this may indicate a bias that may introduced by the sub-sampling or segmentation. Perhaps the authors may have compiled the metagene profiles for the 3´-end control cohort ending at the p(A)-position, which may under-sample or dilute the enrichment for termination factors or T4P beyond.
- P.20 there is a word missing when describing the effect in mNET-seq data, also it would help to give more specific panel references, is this paragraph about S5? It is difficult to follow how the peak of T4P in tandem regions comes as surprise to the authors. Based on (PMID: 28017589) the T4P signal is around the 3´-end of genes, so it would seem justified to interpret the signal for tandem genes as the expected terminating read-through Pol2 transcription.
- P.14 the headings makes it seem as if Pol2 marked with specific modifications accumulate in STIRs, however the data seem to suggest that this enrichment is common to all antibodies. It seems clear that the authors confirmed the expected nascent read-through transcription in the intergenic region between the closely spaced tandem genes.
- Fig3A. X-axis labeling font is too small. In case I read this plot correctly, all proteins are enriched at STIRs (purple). There appears to be not even single region where the signal for the 5´-control (beige) or 3´-control (orange) have higher enrichment values. This specific accumulation pattern appears to be much higher than expected by chance. Do the authors have evidence to consider it a real biological effect that essentially all factors ChIP more to the 5´- regions? It is conceivable this may be an artifact of decreased nucleosome density in STIRs, since promoter and terminators both contribute to NDRs. Perhaps this may increase background binding of ChIP-seq data in these regions. In addition, the differences in n between sample and controls may also affect these analyses since a larger n would likely include more regions without true binding, which could decrease the overall averaged signal.
- For the analyses of histone PTMs it may worth including H4K420me3 since it correlates with repression of the downstream TSSs at MICA (PMID: 29643123).
- Fig.S6. Which Pol2 antibody was used in these mNET-seq experiments? It should be clarified and perhaps compared to the

data in Fig. 5. The paragraph on Spt6/Rft1 lacks a clear hypothesis, what did the authors test here? Do the authors interpret the effects of Spt6 KO as in the plaB treatment where reduced elongation may result in less read-through transcription? It would also help to use the same y-axis scales.
- Fig. 6. There is an impressive amount of work in this figure. The n needs to be clear from the legend. Are analyses based on RNA-seq data? If so, the authors are not in the position to distinguish if signal over the downstream gene corresponds to mRNA initiating from the 5´-SIRT, or may correspond to read-through transcription from the 3´-part of the SIRT configuration. For the selected set there appears to be an upregulation of the downstream gene upon NELF KD, which may be a nice finding to motivate further investigation. Perhaps data from the NELF-AID system (PMID: 32155413) could be used to support this conclusion with an orthogonal approach to deplete NELF that is arguably better suited to reveal the direct effects.
- Is there any experimental data to justify statements on Pol2 recycling in this manuscript? Perhaps some data (PMID: 30150253) aiming to resolve the dynamics of Pol2 transcription could be used to inform if upstream transcription facilitates downstream gene expression by Pol2 recycling. However, addressing this thoroughly will likely require substantial wet lab experiments.

Reviewer #3:

Nissani and Ultitsky present a bioinformatics analysis of closely spaced tandemly oriented genes in the human genome. Such genes are potentially in position where they might be sensitized to transcriptional interference from terminating Pol2 from the upstream gene on initiation at the downstream gene. Analyses suggest that features of intergenic regions, while highly enriched for promoter attributes, are still distinguishable from control promoter sets. Much of the discussion, which is fine for speculation, touches on concept of Pol2 "recycling" to the point that it is prominently featured in the abstract ("Overall, we find that features associated with potential Pol2 recycling rather than those associated with avoidance of transcriptional interference are the predominant driving force shaping these regions"). The arguments supporting this conclusion are somewhat hard to understand and do not appear rise to the level of a conclusion stated in the abstract. It is not clear if there is any positive data to support recycling is what critical feature of intergenic regions vs potential buffering from interference close to promoter. The identification of modified Pol2 accumulating with promoters though diminishing with distance downstream from upstream gene suggests that promoters could have features specifically relevant to slowing or pausing transcription that might otherwise read through or disrupt promoter chromatin. This is a result that potentially deserves greater prominence. Comments for the authors on potential improvements to presentation are indicated below.

1.Analysis for potential GC-related bias in ChIP or other signals. The STIRs are enriched in GC similar to, but higher than, control promoters. If examining the ChIP enrichment for factors in STIRs over control, every single factor is higher in STIRs even if marginally. This potentially suggests there is a technical bias in STIR regions that slightly enriches for binding. Potentially, for ChIP minimally, or elsewhere where it can be examined, this can be assessed or normalized. For ChIP if using the median ratio of all factors for STIRs/controls this would potentially at least adjust for the apparent bias. It might also be important to consider if there is any way, do to increased variance or bias in control regions, or potential issues in the expression matching, if there were bias in mRNA stabilities between STIR and control sets, then expression matched regions might not necessarily be expected to be matched for chromatin binding if factor binding is more expression of synthesis rate than steady state RNA levels.

2. For motifs, is there an analysis that can ask if the marginal enrichment for motifs in STIRs over promoters is predicted solely by the increased GC content?

3. In some cases figures can be confusing or potentially made more accessible to the reader.

4. There are many areas where a more broadly cast net for citation of literature could provide a cleaner context for the reader.

Other comments

Introduction

5. There are currently no citations for the allosteric model of termination. Please consider work of Martinson, H and colleagues.

6. There are studies that suggest in some cases interference might be a regulated phenomenon (work in yeast from Mellor et al (https://pubmed.ncbi.nlm.nih.gov/25407679/) or other cases where it appears that interference has evolved to function as part of gene regulation (Shuman 2020 is cited, but this could be put into context). There are other data (two examples from Arabidopsis https://www.embopress.org/doi/full/10.15252/embr.201949315, https://www.nature.com/articles/s41467-019-12328-w) where it appears that evolution has employed systems to buffer downstream genes from interference that is apparent in mutants. These works and potentially others might benefit the reader. Certainly, the Arabidopsis papers present a strong case that at least in some species, downstream genes might require special protection or be sensitized to interference.

7. p2, middle "The torpedo model connects between nascent RNA.....effect on transcriptional termination (Kim et al. 2004; West, Gromak, and Proudfoot 2004)"

There may be additional papers that describe effects of termination defects on downstream genes, for example, knockdown of XRN3 causes altered expression of some genes and the accumulation of read-through transcripts (Krzyszton, M., Zakrzewska-Placzek, M., Kwasnik, A., Dojer, N., Karlowski, W. and Kufel, J. (2018), Defective XRN3-mediated transcription termination in Arabidopsis affects the expression of protein-coding genes. Plant J, 93: 1017-1031. https://doi.org/10.1111/tpj.13826).

Results

8. p4, middle "closely spaced (<2kb) genes (with the constraints imposed above).. We"

Typo (extra period).

9. Figure 1. Is there any expression level bias between upstream and downstream tandem gene pair, either by mNET-seq or RNA-seq, beyond just asking if they are coexpressed or not, or if there is bias in upstream or downstream when one is expressed and the other is not?

10. Figure 1C. Because of the multiple types of controls used, it can be confusing when terms such as "Minimal STIR-to-Control ratio" is used. What is meant here? Based on potential GC bias and the dominant promoter nature of STIRs, potentially the control here should be the promoter control.

11. p14, bottom "resulting in an overall 6.8 and 8.4-fold enrichment of Pol2 occupancy across the STIR, for the 3' control and promoter control, respectively (see Methods)"

From Figure 5 A and Figure 5S A, it looks like the Pol2 occupancy signal is closer to promoter control. Why does it have more (8.4-fold) enrichment compared with promoter control, but fewer (6.4-fold) enrichment compared with 3' control?

12. p17, middle "with little to no effect on Pol2 in the downstream promoter"

It looks like Pol2 at the downstream promoter is also reduced, the signal is above one before SPT6 degradation then reduced to about 0.6. This is at least not "no effect". What about within the gene or traveling ratio, or expression? Interestingly, spt6 mutants in yeast have reduced transcription interference in the Proudfoot system referenced (https://www.sciencedirect.com/science/article/pii/S0021925820764586) but an Spt6-degron paper from Wolf lab (not sure if cited here, 10.1016/j.molcel.2021.06.016) suggests situation could be more complicated in that Spt6 might also increase readthrough at genes.
)

Discussion
13. p19, bottom and p20, top "Our observation of a slightly...Pol2 pausing at termination sites (Fig. 2D)."
Please check figure callouts- for example, the metagene analysis of MAZ is in figure 2E but it is labeled as figure 2F in this paragraph of discussion.

14. p21, top "G-rich sequences that are also predominantly found near the downstream promoter (Fig. 2E)."

This appears to mean 2D, not 2E.

Figures

15. Figure 2E
Potentially the label of tandem heatmap of metagene analysis could be moved upwards to make it clear given that the label overlaps with the promoter control heat map. Potentially label could be horizontal for this heatmap.

16. In some cases it is not apparent why P values are different over individual control regions where it seems that STIR signal is very similarly higher than either control region but one shows significance and another doesn't. This is sort of hard to intuitively understand.

During our pre-decision cross-commenting process, Reviewers #2 and #3 made additional comments, which I have included below.

Reviewer #3

1) It is true that many in field use "TTS" to represent polyA site. The labeling should change to indicate that. However, since

termination that might result in interference will be obligately downstream of the polyA site, it should be fine to use polyA site as proxy for measuring intergenic distances in this study.

2) If nascent read through in intergenic regions is difficult to discern, authors might provide caveats to the observation that modified Pol2 is found near downstream promoters. The result for S2P and T4P is potentially striking enough that even with caveat that it is difficult to tell where it originated from, it should be reported. An indirect argument would be that this signal appears to diminish with distance from upstream gene, suggesting a possible connection.

3) I have less of a concern about potential lncRNA or other upstream transcription confounding controls, only because this would cause underestimation of the enrichments they observe, not over estimation, but it is a good point to be careful about.

4) The authors might additionally consider asking if downstream genes of STIR pairs have greater or lesser bidirectional transcription- an additional difference could be that they are evolved to have enhanced directionality on the downstream side to diminish divergent transcription that could be antisense to the upstream gene. Additionally, it might be useful to show mNET seq data for the bottom strand for all the different Pol2 modifications, this should allow a more clear view of what is going on here.

Reviewer #2

A comment on the cross-comment points 2) and 3). The CTD signal mentioned in 2) could for instance stem from the missed transcription units mentioned in point 3). A correct segmentation with the data and know-how of 2021 will be necessary.

Point-by-point response to reviewers for "Unique features of transcription termination and initiation at closely spaced tandem human genes" by Nissani & Ulitsky

Reviewer #1

In the present study, Nissani & Ulitsky addresses the interesting question of how transcription termination of a gene can impact transcription of a closely spaced downstream gene in human cells. Specifically, the authors investigate whether intergenic regions between closely spaced and co-expressed tandem genes (distance less than 2 kb) possess specific features. To address these questions Nissani & Ulitsky re-analyzed available genome-wide ENCODE data (RNA-seq, ChIP-seq) and mNET-seq data. In a first step, the authors systematically identified gene pairs and found that co-expression was most common in closely spaced (<2 kb) gene pairs. The authors then focused primarily on the 188 closely spaced and co-expressed tandem genes that they identified in human HepG2 cells. Next, the authors investigated if the short intergenic regions between close tandem genes, called STIRs, are characterized by specific features. Notably, this analysis indeed revealed distinct characteristics including sequence features. The authors provide strong evidence for an enrichment of G nucleotides in STIRs and uncovered G-rich sequence motifs. Furthermore, the authors show that these regions are characterized by distinct protein binding events, such as of AGO1 and AGO2, and also provide some evidence that RNA polymerase II has a specific CTD phosphorylation pattern at STIRs.

The computational analyses were carefully performed and the obtained results of this work are exciting. A clear strength of this work is the identification of specific features of STIRs in human cells. The most exciting finding at least for me is the discovery of sequence features including G-rich motifs that are enriched in STIRs. Overall the manuscript is excellently written and main conclusions are supported by the data that is provided. Furthermore, the authors did an exceptional job in creating high-quality figures that make it easy for readers to immediately grasp key findings. This study also opens up interesting future research directions such as to further functionally and mechanistically characterize the sequence elements that the authors uncovered in this study. I have no doubts that the findings of this work will be of broad interest for researchers in the transcription field and to researchers of other disciplines who have an interest in transcription regulation.

Major comments:

(1) The authors analysed RNA-seq data upon knockdown of 245 different factors and focused on factor depletions upon which the expression of the up- or downstream genes were differently affected. The authors then almost exclusively focused on the

observations obtained upon NELF-E/NELF complex knockdown. Although, I agree that this complex is of particular interest in this context, the list of factors that is provided in Table S10 also contains other interesting factors that could be mentioned and discussed in the main text.
Please clearly state in the main text how the up- and downstream genes are affected upon NELF-E knockdown. Please also simplify Table S10 (now EV11), if possible, so that the reader immediately recognizes how the expression of the up- and downstream genes are altered upon the respective factor knockdown.

We now revised the sentence in the text referring to NELF-E:
*"This analysis highlighted Negative Elongation Factor Complex Member E (NELF-E), the knockdown of which led to an increased expression of the downstream gene in a tandem pair, while not affecting the upstream gene (**Fig. 6** and **Table EV11**)"*

We also revised and simplified Table S10. Regarding discussing additional factors, we already mentioned additional factors SMN1 and XRN2, whose knockdown leads to up-regulation of the downstream tandem genes, and now also discuss U2AF1 whose KD leads to down-regulation of tandem downstream genes:

*In addition to NELF-E, we found SMN1 and XRN2 KD as having similar, yet less robust effects of upregulation of the downstream and not the upstream tandem gene (**Fig. 6A** and **S4F-G** and data not shown). Intriguingly, SMN was previously suggested to play a role in R-loops resolution (Jangi et al, 2017; Zhao et al, 2016). Fewer KD experiments negatively affected just the downstream gene. Notably, KD of U2AF1 had this effect in both K562 and HepG2 cells (**Fig. 6A**), and mutations in U2AF1 in myelodysplastic syndromes are associated with reduction of Pol2 pause release and R loop accumulation (Chen et al, 2018; Nguyen et al, 2018).*

(2) The authors propose a model according to which the STIR region may represent a 'preparation area' for a new transcription cycle of the downstream gene, leading to a recycling of the Pol II that has transcribed the upstream gene. As presented, this model is primarily based on the Pol II T4P occupancy data. If both transcription cycles are coupled, I would have expected that rapid depletion of the key Pol II transcription factor SPT6 not only impacts Pol II density at the STIR region, but also as a consequence in the promoter-proximal region of the downstream gene. However, this seems not to be the case. Although rapid SPT6 depletion induces a collapse of Pol II over the STIR region, the Pol II density in the promoter-proximal region of the downstream gene seems not to be affected (Figure S6A, B). Other potential limitations that need to be considered in this context are: (1) it is likely that Pol II and its CTD isoforms which are detected in the narrow STIR region are a mixed population of terminating (upstream gene) and promoter-bound/initiating Pol II (downstream gene). (2) This study relies on

bulk data which provides an average view on Pol II transcription. It remains unclear if the upstream and downstream genes are indeed expressed at the same time in the same cell. This similarly affects the interpretation of the Pol II occupancy data. It would be helpful to clarify this a bit more when presenting the model.

Following this comment, as well as comments from the other reviewers, we have now revised Figure S6A (now Figure EV4), to have the same y axis in both control and SPT6-AID cells, which shows that there is indeed a reduction in the promoter region in both STIRs and control promoters.

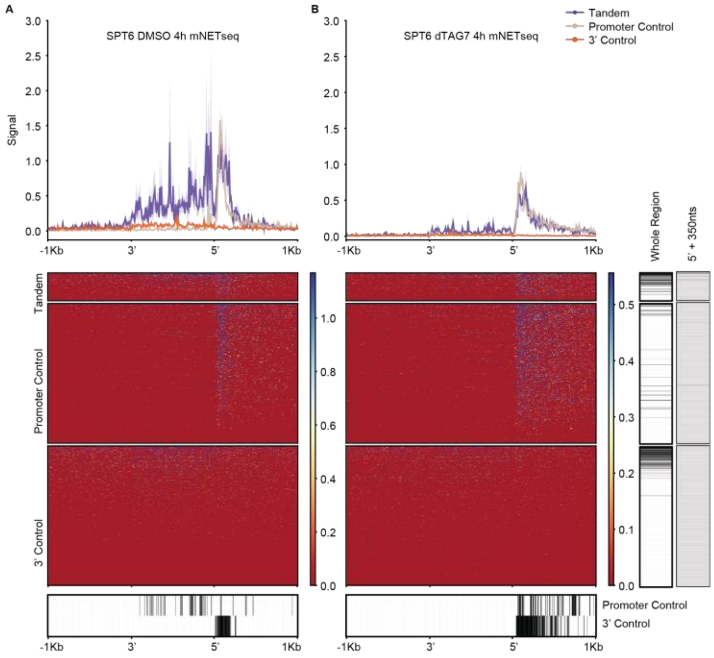

Figure EV4A-B:
(A) Metagene analysis (top) and corresponding heatmap (center) showing mNET-seq median signal following control (DMSO) treatment in K562 co-expressed STIRs and flanking regions and in their controls. Bottom heatmap shows the corrected P-value of binned paired Wilcoxon rank-sum test. Data from (Žumer *et al*, 2021), using the CMA601 anti-CTD antibody as in **Figs. 5 and S2**.
(B) As in (A), for K562 cells 4h after treatment with SPT6 dTAG7. Centered heatmap is ordered by the gene order in the heatmap of (A). Right heatmap is the corrected paired Wilcoxon rank-sum test per gene before and after treatment. Proportion of significantly changed signal for genes following the KD treatment was tested between the group of tandem genes and each control group and was significant in both cases ($P=4.4\times10^{-31}$ and $P=1.1\times10^{-9}$ for the promoter or the 3' control, respectively).

We also changed the text accordingly:

*This accumulation was strongly reduced upon targeted degradation of Spt6 (**Fig. EV4A-B**), with a smaller effect on Pol2 in the downstream promoter.*

We agree with the reviewer that all the data we analyze is coming from a large population of cells, and it is difficult to know if transcription events occur at the same time or at consecutive time periods in individual cells. We now address this in the Discussion:

Specifically, since we analyze bulk data from a large number of cells, it is unclear to what extent the transcription events of tandem genes occur concurrently or in short temporal succession. The combination of methods enabling single-cell metabolic labeling with those for single-cell chromatin occupancy can be particularly useful for addressing this question in the future (Erhard et al. 2019; Bartosovic et al. 2021).

Minor comments:

(1) On page 2 (Introduction) the authors describe the two models of transcription termination: the allosteric and the torpedo model. Please add the corresponding references.

We added references to both models.

(2) On Page 3 (Introduction) the authors mention that Pol II CTD S2P and T4P have been linked to transcription termination. The authors should mention that Pol II CTD Y1P has also been linked to transcription termination in yeast (PMID: 22745433) and mammals (PMID: 29304333).

We added these references:

"In addition, phosphorylated Threonine-4 (T4P) has also been correlated with termination regions (McCracken et al. 1997; Heidemann et al. 2013; Hsin and Manley 2012; Schlackow et al. 2017; Nojima et al. 2018), and Tyrosine-1 (Y1P) has been linked to termination in yeast (Mayer et al. 2012) and mammals (Shah et al. 2018)."

(3) Figure 1A: please mention the data source in the figure legend.

We now mention that the data are from the ENCODE project.

(4) Figure 1G: can you please clarify in the figure legend if "3' Upstream gene" means cleavage and polyadenylation site (CAS).

We have clarified the legend, and now use the "CPA site" terminology for the 3' ends to make the text clearer.

(5) The authors identified G-rich sequence motifs in the short intergenic region (Figure 2A). Do these motifs have any similarity to known transcription factor binding sites (except from MAZ and Sp1)?

Following the suggestion of the reviewer, we used the Tomtom tool from the MEME suite alongside the 2022 JASPAR vertebrates motif dataset with motifs for 838 factors, 3 of these intersected between GGGGCGGG and GGGGCGGGGSC (requiring both E- and q-value < 0.05): MAZ, SP5 (a member of the Sp1 family), and ZNF138 (a poorly studied TF). We now mention this in the text.

(6) Please specify in the text if the antibody that was used to obtain total Pol II mNET-seq (CMA601) is directed/or not directed against the Pol II CTD (to avoid any CTD bias).

The CMA601 antibody, previously used by the Proudfoot and the Kimura labs is directed against total Pol2 CTD and detects both the phosphorylated and the unphosphorylated CTDs. We now clarify this in the text, with the corresponding citations:

> *Therefore, it is unexpected that we find extensive Pol2 signal in STIRs, compared to the control genes, in the "total Pol2" mNET-seq data (**Fig. 5A**, using anti-CTD CMA601 antibody, recognizing both phosphorylated and unphosphorylated CTD (Nojima et al, 2015; Stasevich et al, 2014)). Further, this trend is seen when examining Pol2 with all the different CTD modifications (**Fig. 5B-F and Fig. S2**), including high levels of T4P signal downstream of the downstream TSS while being almost at background levels in the corresponding regions in both controls.*

(7) Figure 5: please mention the number of replicates in the figure legend.

We now mention in the figure legend that a representative replicate is shown. We found that replicates look very similar in this analysis (see Reviewer Figure below), and since a large number of plots is already shown, prefer not to add additional panels for replicates.

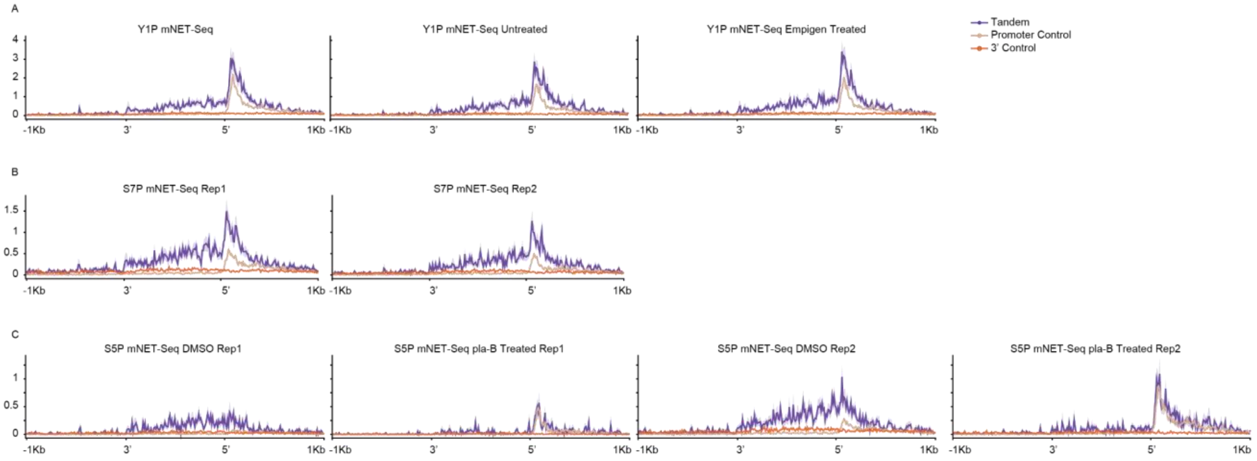

Reviewer Figure 1: Examples of replicates of treated and non-treated experiments based on data from (Schlackow et al. 2017) (as shown in the paper) for (A) Y1P with or without Empigen treatment, (B) S7P and (C) S5P with or without pla-B treatment. Replicate number is indicated above each panel.

Reviewer #2
The authors present an extensive analysis of genomics data focused on a few hundred pairs of closely spaced human genes oriented in tandem. They identify several genomic features enriched in these genomics intervals. Some perturbations of transcription correlate with preferential effects on the expression of the downstream gene. The authors conclude that features associated with potential Pol2 recycling may the predominant driving force shaping these regions.

The quality of data analyses is very good. The writing is clear and easy to follow. The figures are well-crafted for the most part help to highlight the key finding. In these respects the manuscript is impressive since it presents an analysis of a large data volume in a coherent style. A satisfactory systems-level understanding of gene regulation in the tandem configuration was arguably not reached. The manuscripts offers no original laboratory experiments to test any of the correlations, which likely limits the scope, novelty and impact of this manuscript. The conclusions on Pol2 recycling and transcriptional interference are insufficiently supported. On the other hand, many relevant genomics resources have been exhausted to address tandem gene regulation, and so the manuscript may be of interest to genomicists. Comments to improve the manuscript are listed below.

- The authors identified closely spaced tandem genes. In general, the presence of un-annotated and cryptic transcripts (lncRNAs, or transcripts only in nascent Pol2 data) is a potential confounding factor for the interpretation of various signals and signatures. Perhaps I missed it, but I did not find a paragraph in the text and method how this confounding factor was handled. Additional transcription units in SIRTs would have the effect that the selected tandem transcription units may in fact not be tandem, since un-annotated transcripts may be present in the areas. Likewise, others genes that were excluded may in fact be tandem with a cryptic non-coding transcript. Performing the segmentation properly is a complicated task, but it may particularly straightforward to address if the downstream promoters give rise to divergent lncRNA transcription using various resources for nuclear exosome RNA decay factor perturbations, for example (PMID: 32710631, 27455346, 32075771). If so, the upstream transcripts would actually be tail-to-tail with a divergent lncRNA. Essentially, the classification of tandem genes may be confounded by other transcripts that would result in a different segmentation if tandem transcripts (coding and non-coding) were considered.

We agree with the reviewer that STIR units, as well as other promoters and 3' ends are likely to have complex transcriptional behavior. The major challenge is that because of the quantitative nature of transcription, depending on the thresholds used, noncoding transcripts can be found near virtually all promoters, and in both directions, and so it is

exceptionally difficult (or impossible) to obtain a ‘complete’ transcriptome annotation. Therefore, we chose to focus on a relatively conservative set of protein-coding genes, in particular their longest annotated isoforms. While it is certainly possible that some of the STIRs contain additional transcriptional units, we now show these are rare/lowly expressed enough to not substantially affect our results. As mentioned by one of the reviewers in the exchange between the reviewers, if tandem noncoding transcripts are found in our “control promoter” set, this should strengthen our observations that we report rather than weaken them.

We now show the levels of nascent transcription on both strands from ChRNA-seq data in HeLa cells in Figure EV1C, where it is evident that while there is some additional transcription on both strands beyond the gene boundaries that we consider, it is at substantially lower levels than the transcription of the main gene units:

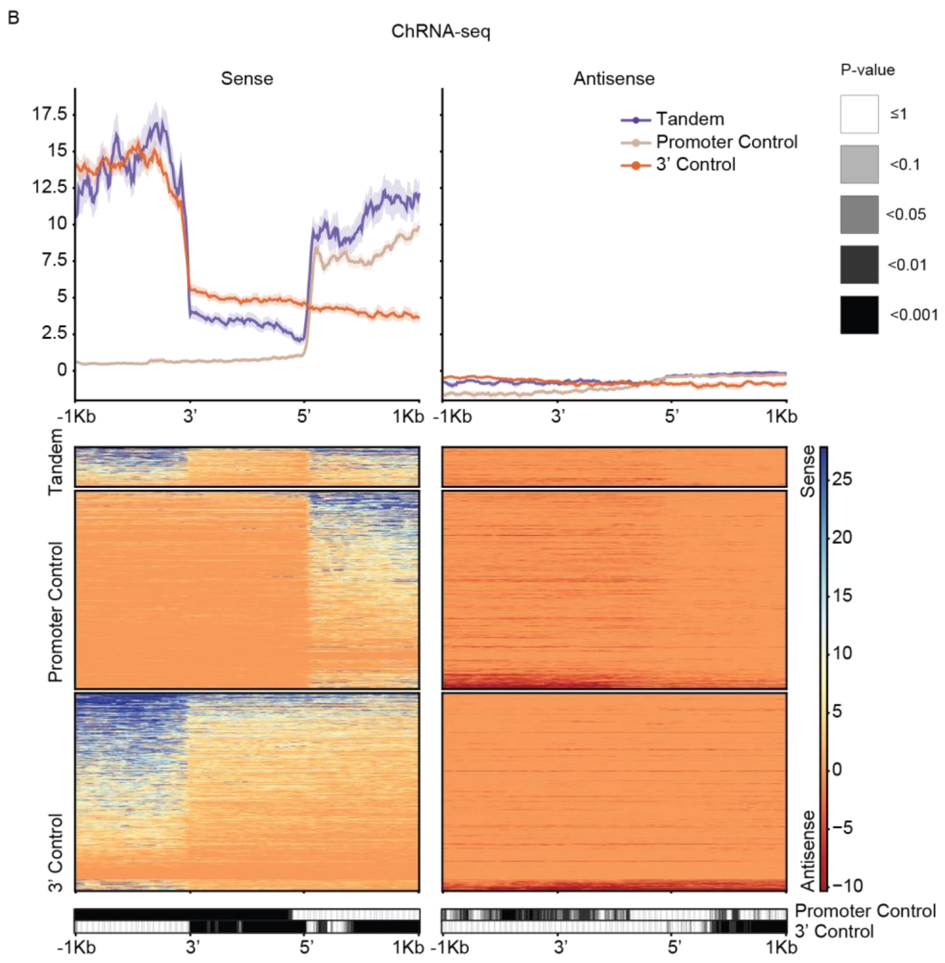

Figure EV1 (C) (Now EV1C) Sense and antisense metagene profile (top) and heatmap (center) of median and standard error chromatin RNA-seq signal based on data from (Schlackow et al. 2017). Profile of the STIRs in HeLa cell line is plotted in between the CPA site of the upstream gene (5’) to the TSS of the downstream tandem gene (3’) and in 1kb flanking sequence. For the control genes, the TSS of promoter control genes are aligned to the TSS of the downstream gene (beige), and the CPA sites of 3’ control genes are aligned to the CPA sites of the upstream gene (orange). Negative values indicate transcription on the antisense strand. Bottom plot shows the binned Bonferroni-corrected paired Wilcoxon rank-sum test P-value heatmap.

Related to this point, we agree that a full-transcriptome segmentation would be useful for extending our analysis, but because of the same reasons stated above, we argue that it is exceptionally difficult in the gene-dense regions that we focus on here, without the availability of particularly deep long-read RNA-seq data. In the gene-dense parts of the genome we expect to find a multitude of transcriptional units that are overlapping each other, expressed at different levels (see e.g., https://www.nature.com/articles/ng.3616), and exhibiting variable stability levels. In this complex environment, it is exceptionally difficult to perform our analysis focused on interplay between tandem units. Therefore, we prefer to focus on the simpler question of the interplay between robustly-expressed protein-coding transcripts, and leave the more complex segmentation-based analysis for future studies, that will be fueled by long-read RNA-seq data.
We now discuss this topic in the text:

*In our analysis, we focused on regions separating well-expressed protein-coding genes, but pervasive transcription of mammalian genomes leads to many additional transcripts produced near most human promoters, mainly in an antisense orientation to the protein-coding genes (Chen et al, 2016). Many of these transcripts are rapidly degraded, which results in a wide range of expression levels. Inspection of RNA-seq read coverage on both strands shows that antisense transcription is also present at our STIRs, but at much lower levels than the transcription of the sense strands, and, within the first half of the STIR, at levels lower than that of control promoters (**Fig. EV1C**). Transcription at downstream promoters in tandem pairs thus appears to be more unidirectional than in the controls. In some cases, there could also be additional transcripts on the sense strand that we are not considering, which can affect the boundaries of the STIRs and potentially invalidate some of our control genes, but inspection of ChRNA-seq coverage suggests that such transcripts are either rare or very lowly expressed (**Fig. EV1C**). Full-transcriptome segmentation into gene units that are agnostic to the protein-coding potential, as performed in some species (Ivanov et al, 2021), and which would benefit from long-reads, would potentially allow a more accurate analysis of the cross-talk between closely spaced transcriptional units. In any case, the wide range of expression levels of the different transcripts will retain the challenge of how to define "intergenic regions".*

- Predominantly in early parts of the manuscript a focus appears to be on transcriptional interference. Since the mechanism of transcriptional interference is not restricted to mRNA-mRNA pairs, this segmentation is too crude to justify statements on this mechanism. Either the writing needs to reflect this, or a higher quality segmentation needs to be presented that relies on a fully updated genome annotation including short and long ncRNA transcription units in these regions that may be transcribed on both

strands. Data-driven genome annotation in other systems may serve as inspiration here (PMID: 34058980).

As mentioned above, we prefer to defer the full segmentation to future studies based on deep long-read sequencing due to the complexity of the segmentation in mammalian genomes, where there are typically many overlapping and mostly lowly-expressed transcription units. We now show that when examining chromatin-associated RNA there is very limited transcription on the same strand immediately upstream on the annotated TSSes, and that splicing of introns in the downstream gene appears to be robust. We also analyzed nano-COP long-read RNA sequencing data and found limited evidence for readthrough transcripts connecting the upstream and the downstream genes:

*We considered the possibility that read-through transcription through the STIR might occasionally generate fusion transcripts which may confound our analysis. Such read-through has been observed upon different perturbations in human cells (Arnold et al, 2021; Vilborg et al, 2017). Several lines of evidence suggest that these events are exceedingly rare in unperturbed cells. First, examination of ChRNA-seq data of nascent transcripts showed that read coverage immediately upstream of the TSS of the downstream gene is reduced to almost baseline levels (**Fig. EV1C**). Second, splicing efficiency of the downstream gene (which might be reduced if parts of it correspond to a long 3' UTR of the upstream gene) was rather indistinguishable from that of controls, including in the first intron, where splicing efficiency was even greater in tandem genes compared to controls (**Fig. EV1D-E**). Third, when considering long-read nano-COP data (Drexler et al, 2020), of the 20,925 reads mapping to either the upstream or the downstream gene in a tandem pair, only 122 (0.6%) overlapped exons of both genes, and none contained spliced-out introns in both genes. For only 1.8% of the 131 tandem gene pairs for which both genes had at least 10 nano-COP reads, there were at least 2 reads overlapping both genes. In contrast, 90% of the reads that overlapped the CPA site of the upstream gene overlapped at least 50 nt of the STIR, and 57% of the upstream genes had at least 2 reads aligning to both the gene and the STIR. These results suggest that whereas Pol2 that transcribes the upstream gene continues to transcribe after the CPA site, as expected, it very rarely extends the upstream transcript beyond the TSS position of the downstream gene.*

We now also discuss the option of noncoding transcripts confounding our results in the text, as well as tone down our statements on transcriptional interference.

Nevertheless, when the distance between adjacent genes is short, there is no evidence that tandem genes are less likely to be co-expressed, which could be expected if transcriptional interference was posing a substantial obstruction to transcription from downstream promoters (in which case we would expect to see selection for avoidance of co-expression, specifically for tandem pairs), although such interference can still come from unannotated ncRNAs that we do not consider here.

- Conclusion Page 4 bottom. It may indicate a logical flaw to expect that transcriptional interference poses a substantial obstruction to transcription from the downstream promoters. In fact, the downstream gene is often expressed at high levels, yet no functional initiation occurs (PMID: 6329902, 15175754, 30707695 and many others). The method to measure expression, it appears to be RNA-seq, should be explicitly stated in the text. In any case, unless the authors rely on appropriate methods here that resolve Pol2 initiation in these regions and have information on the nature of the overlapping transcripts, it is inadequate to make statements about the mechanism of transcriptional interference.

We agree with the reviewer that the presence of transcriptional readthrough would complicate our results. However, we observe that there is little to no nascent transcription immediately before the downstream promoters in STIRs, as we now show in **Fig. EV1C** pasted above. We also find that the downstream genes in tandem pairs are as efficiently spliced as control genes without an upstream tandem gene (**Fig. EV1D**), including specifically at the first intron, which argues against a substantial signal coming from “extended 3'UTRs” of the upstream genes. Lastly, we analyze long-read RNA-seq and find that fused transcripts mapping to exons of both the upstream and the downstream gene are rare, as discussed in the. We now updated the text to reflect the possibility of read-through transcripts confounding our results and to present the long-read sequencing, all as described above.

- Page 7, end 1st paragraph and 1G legend. How were the TTS positions determined? Is that information based on mNET-seq data or are the authors referring to poly-(A) positions here? The poly-(A) position is not the equivalent to the TTS. Similar in S1, since most standard genome annotation lack the important read-through tail that could give the range of TTSs, it looks like the red lines are probably p(A) - TSS distances. Using TTS-TSS distances would be much more appropriate, at least for an investigation of transcriptional interference.

We agree with the reviewer that our use of the TTS term was inaccurate, as these are largely not annotated, we now switched throughout the manuscript to the use of the “CPA site” term, referring to the 3' end position, that is typically based in RefSeq annotations on the genomic position of the Poly(A) tail start from full-length cDNA or EST data.

Perhaps an analyses of POINT or nano-COP (PMID: 33735606, 31839405) may offers some insight into where nascent transcription signal in SIRTs represents terminating transcription from the upstream genes, un-annotated nascent transcripts or cryptic initiation of the downstream genes.

As mentioned above, as suggested, we now analyzed nano-COP data and quantified the extent to which transcriptional fusions between the upstream and the downstream genes may occur, and found such events to be exceedingly rare. As expected, we do find cases of read-through of transcription from the upstream gene into the STIR region.

- P.20. Related point, "During transcription termination, the current model is that transcription continues for a few hundreds to several kbs post the TTS before the polymerase is released from the DNA ... " there is some confusion. TTS needs to be replaced with cleavage site where the p(A)-tail is added in this sentence. Transcription continues beyond this position, so it is by definition not terminated yet. The TTS is the termination site, which is often missing in genome annotations but at least a read-through tail that informs on TTSs is annotated for some genomes (PMID: 34058980).

As mentioned above, we have now switched to using the "CPA site" terminology, as actually TTS sites are typically not well defined and not annotated in the human genome.

- ChIP-seq data representations in Fig2EF, 3D-G, 4A. The genomic regions used as controls need to be explicitly defined. Presumably these correspond to the same groups as for the intron analyses on top of p.7, but it is not defined in the legends. Also, what justifies the choice of 5x more control regions than target STIRs? Selecting the same n would be more intuitive to allow for equal noise effects in the control regions.

We use the same set of controls in Figure 2 as throughout the manuscript, and now clarified this in the legend of Figure 2 and on page 7 (*"These controls were used in all subsequent analyses."*). We chose to use 5X as many control genes as the set of STIR genes to reduce variability among the controls, and to increase the power we have when comparing regions and since it was possible, as there are many more genes without a tandem gene pair. This should not affect any of the conclusions, for example:

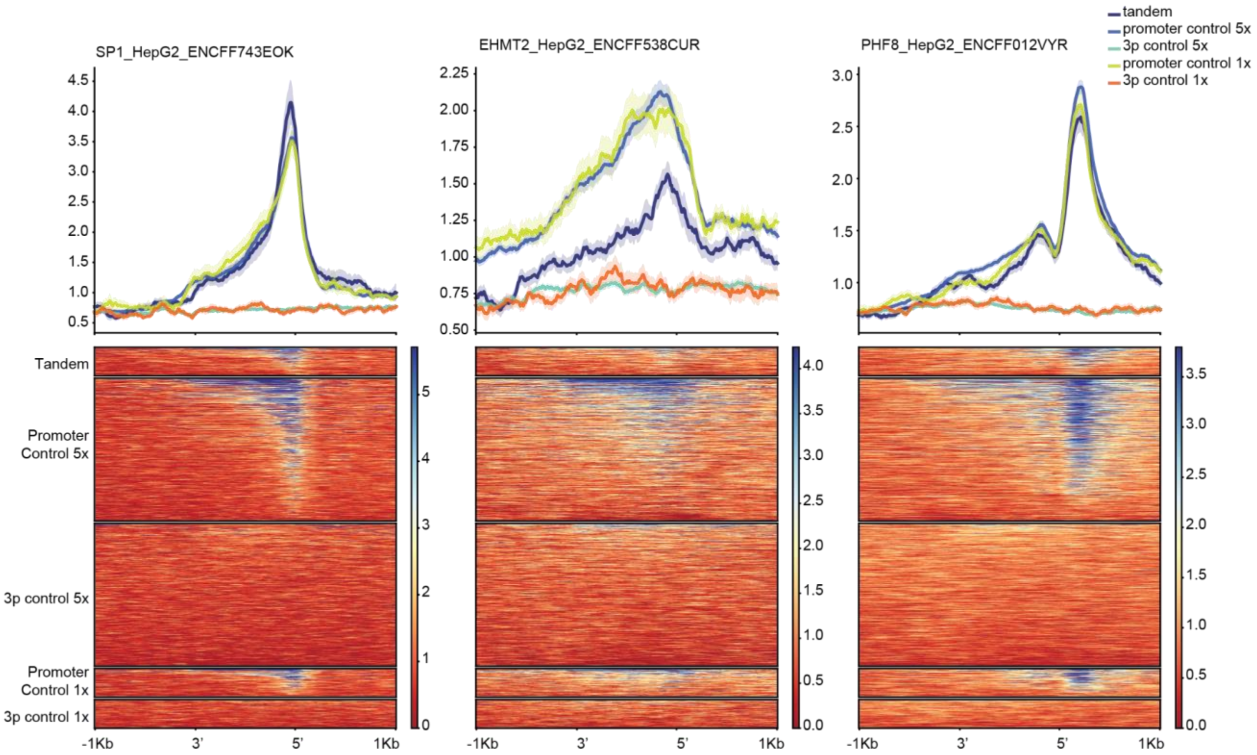

Reviewer Figure 2: Examples showing the profile (top) and heatmap (bottom) analysis signals for 3 transcription factors (SP1, EHMT2, and PHF8) showing either a single control gene (yellow and orange) per tandem pair or 5 control genes (as shown in the paper, blue or teal) per tandem pair (appear in dark blue).

- The factors in Fig2EF, 3D-G, 4A, S3D, S4A,B,D appear to ChIP to the 5´-gene. It would be good to show that some factors specifically enrich for the 3´-end as control, otherwise this may indicate a bias that may be introduced by the sub-sampling or segmentation. Perhaps the authors may have compiled the metagene profiles for the 3´-end control cohort ending at the p(A)-position, which may under-sample or dilute the enrichment for termination factors or T4P beyond.

Most of the factors profiled by the ENCODE project so far map to the 5' ends of genes, and the factors that map to STIRs are not an exception. There are overall few factors that ENCODE profiled that are enriched at 3' ends. We now show this explicitly, by comparing our “promoter control” and “3’ control” gene sets, and counting for each ChIP-seq dataset the number of overlaps with each control (note that this analysis does not consider tandem genes at all, but rather “standalone” promoters and 3' ends). This analysis is presented in Figure EV2A-B:

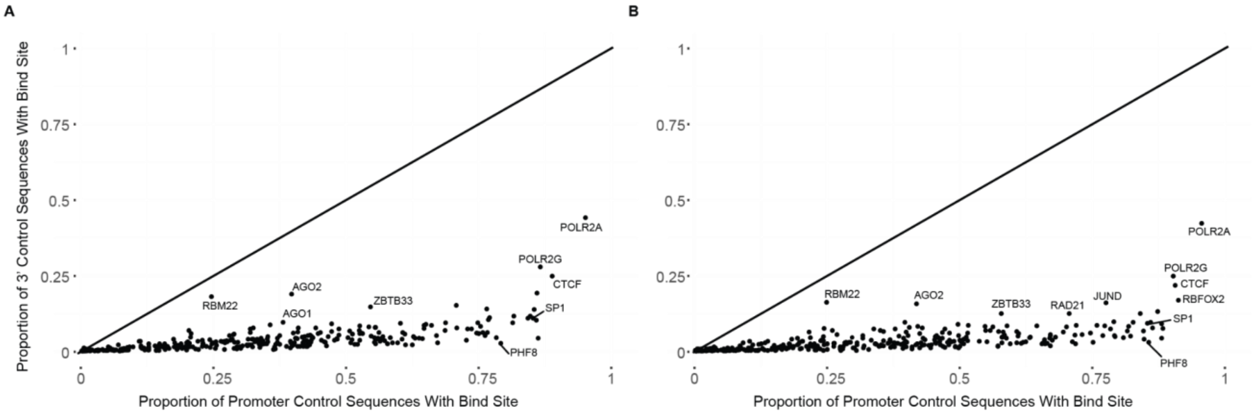

Figure EV2A-B (A-B) Scatter plot showing the proportion of ChIP-seq–based binding sites at promoter- (x-axis) or 3'- (y-axis) control sequences in HepG2 (A) or K562 (B) cells. Marked are selected proteins of interest or proteins with relatively high proportions in both axes. Black diagonal line represents equal proportions.

We also now added Figures 3A (right panel) and EV2C (right panel) that present independently the enrichment of STIRs relative to each type of controls. These plots show that whereas most factors are generally enriched at both standalone and tandem promoters, there is a small select subset of them that preferentially binding in STIRs, and which are highlighted in **Figure 3A and EV2C.**

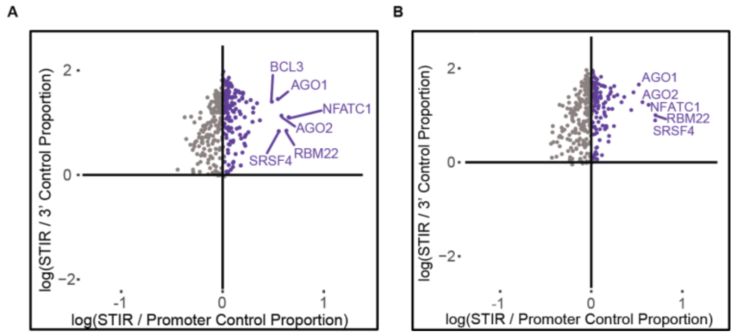

Figures 3A and EV2C (right panels): Scatter plots showing the log-transformed proportion ratio between binding sites at STIRs or at either control, in purple are proteins enriched in STIRs over both types of controls. Indicated are several notably enriched proteins in (A) HepG2 or (B) K562 cell lines.

- P.20 there is a word missing when describing the effect in mNET-seq data, also it would help to give more specific panel references, is this paragraph about S5? It is difficult to follow how the peak of T4P in tandem regions comes as surprise to the authors. Based on (PMID: 28017589) the T4P signal is around the 3´-end of genes, so it would seem justified to interpret the signal for tandem genes as the expected terminating read-through Pol2 transcription.

We have revised this paragraph and added references to specific panels in Fig. 5. It is not surprising that the peak is found near the 3' end of the upstream gene, but rather surprising that this peak is much stronger than that observed at “3' control” genes that

are matched for expression levels with the “tandem upstream” genes, yet display much lower levels of T4P-modified Pol2 occupancy:

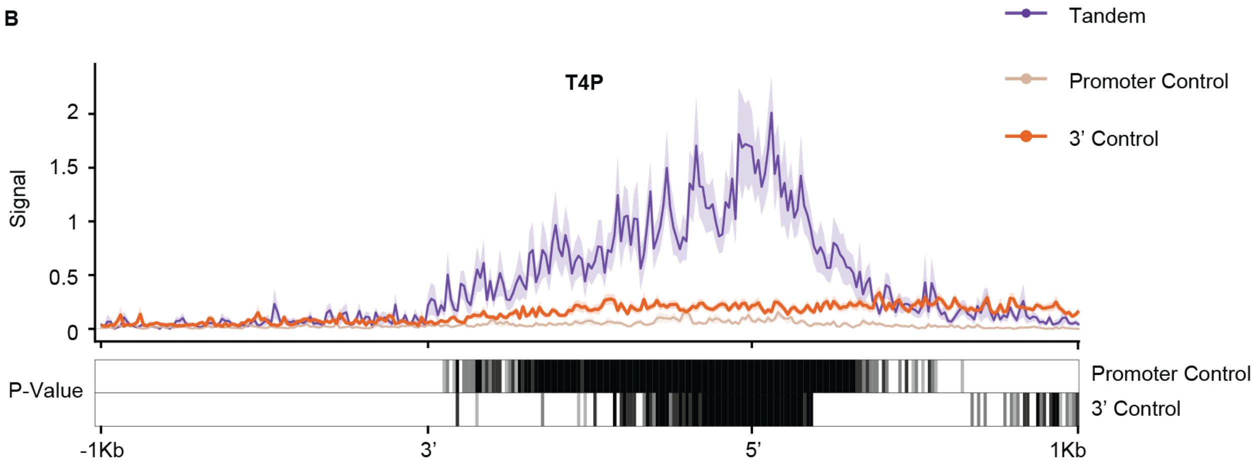

Figure 5B: Metagene analysis (top) showing the different median Pol2 carboxy-terminal domain T4P modification occupancy signal at the sense strand of STIRs or controls and their flanking regions. Bottom are the respective corrected paired Wilcoxon rank-sum test P-values. Data from Schlackow *et al.*, 2017.

We have emphasized this further in the text:

*Based on the current model of transcription termination, transcription continues for a few hundreds of bps to several kbs post the CPA site before Pol2 is released from the DNA. This is thought to be accompanied by the T4P CTD modification of Pol2. Considering this model, we expect that the Pol2 T4P signal, other Pol2 modifications associated with termination, and the general Pol2 signal to be similar at the termination sites of all genes, irrespective of their proximity to another transcriptional unit. Therefore, it is unexpected that we find extensive Pol2 signal in STIRs, compared to the control genes, in the “total Pol2” mNET-seq data (**Fig. 5A**, using anti-CTD CMA601 antibody, recognizing both phosphorylated and unphosphorylated CTD (Nojima et al, 2015; Stasevich et al, 2014)). Further, this trend is seen when examining Pol2 with all the different CTD modifications (**Fig. 5B-F and Fig. S2**), including high levels of T4P signal downstream of the downstream TSS while being almost at background levels in the corresponding regions in both controls. Particularly striking is the prominent peak of T4P-modified Pol2 at the downstream promoter, whereas such Pol2 is rarely found at control promoters (**Fig. 5B and S2G**).*

- P.14 the headings makes it seem as if Pol2 marked with specific modifications accumulate in STIRs, however the data seem to suggest that this enrichment is common to all antibodies. It seems clear that the authors confirmed the expected nascent read-through transcription in the intergenic region between the closely spaced tandem genes.

As discussed above, it is not surprising that Pol2 is enriched beyond the CPA site, but rather that the enrichment is much higher in STIRs than downstream of expression-matched control genes that are not followed by another promoter. The reviewer is correct that we see enrichment for various modifications, and so we removed the "marked with specific modifications" statement from the section header and from the abstract.

- Fig3A. X-axis labeling font is too small. In case I read this plot correctly, all proteins are enriched at STIRs (purple). There appears to be not even single region where the signal for the 5´-control (beige) or 3´-control (orange) have higher enrichment values. This specific accumulation pattern appears to be much higher than expected by chance. Do the authors have evidence to consider it a real biological effect that essentially all factors ChIP more to the 5´- regions? It is conceivable this may be an artifact of decreased nucleosome density in STIRs, since promoter and terminators both contribute to NDRs. Perhaps this may increase background binding of ChIP-seq data in these regions. In addition, the differences in n between sample and controls may also affect these analyses since a larger n would likely include more regions without true binding, which could decrease the overall averaged signal.

As mentioned above, we now show the propensity of all the factors profiled by ENCODE to bind at control promoters and 3' regions and also show all the enrichment values relative to both kinds of controls, whereas before we only showed factors that were enriched.

- For the analyses of histone PTMs it may worth including H4K20me3 since it correlates with repression of the downstream TSSs at MICA (PMID: 29643123).

We now analyzed data from H4K20me3 ChIP-seq in IMR90 cells (see **Reviewer Fig. 3** below), but did not observe any particular enrichment or trend beyond overall depletion of histones in promoters. Since it is difficult to evaluate the quality of the dataset, and we already show many other histone marks, we preferred not to include this analysis in the text.

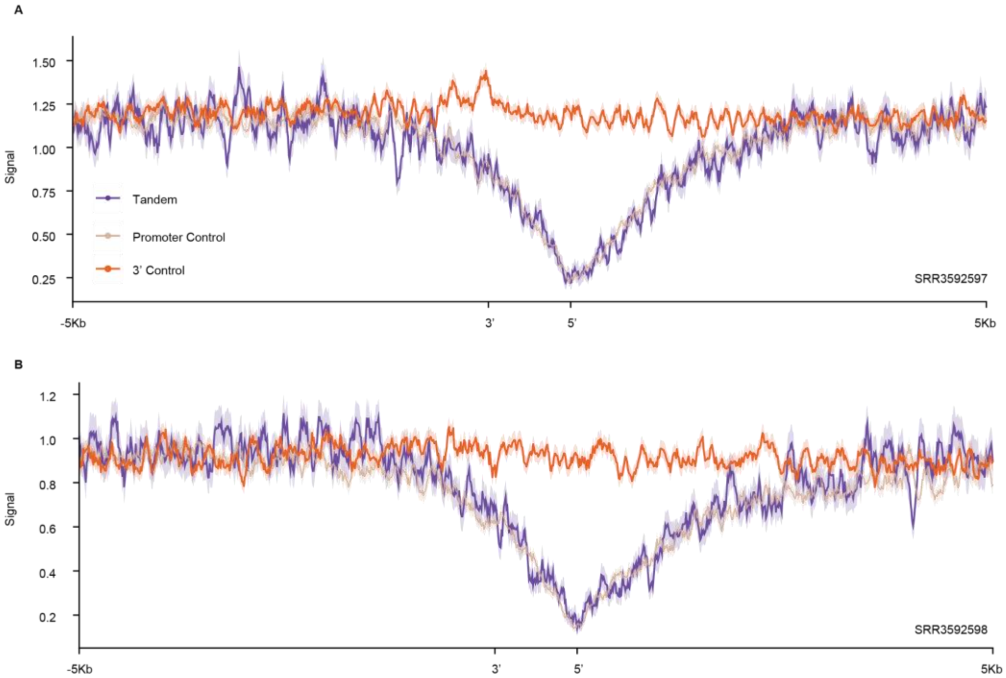

Reviewer Figure 3: Metagene plots of ChIP-Seq signal of H4K20me3 histone modification for two replicates based on data from (Nelson et al. 2016), experiment was performed in IMR90 cell line.

- Fig.S6. Which Pol2 antibody was used in these mNET-seq experiments? It should be clarified and perhaps compared to the data in Fig. 5. The paragraph on Spt6/Rft1 lacks a clear hypothesis, what did the authors test here? Do the authors interpret the effects of Spt6 KO as in the plaB treatment where reduced elongation may result in less read-through transcription? It would also help to use the same y-axis scales.

These data are based on the same anti-CTD CMA601 antibody as the Proudfoot lab data, and we now clarify this in the legend of Fig. S6 (**Figure EV4**). The goal of this analysis was to show that treatments that result in a reduction in transcription of the upstream gene also eliminate the Pol2 found in the STIRs. We now edited this paragraph based on the comments in the reviewers:

*As further evaluation of the role of upstream gene elongation in the accumulation of Pol2 in STIRs, we also examined the consequences of loss of Spt6 and Rtf1, two factors reported to separately control Pol2 progression. We used mNET-seq data from K562 following Spt6 and Rtf1 depletion from (Žumer et al, 2021) and examined Pol2 distribution within STIRs (**Fig. EV4**). Spt6 was shown to assist progression of elongating Pol2 through nucleosomes, and its depletion was shown to have little to no effect on Pol2 accumulated at promoters (Žumer et al, 2021). Indeed, data in K562 recapitulated the pattern we observed in HeLa cells, showing strong accumulation of*

*Pol2 in STIRs in control conditions (DMSO-treated cells). This accumulation was strongly reduced upon targeted degradation of Spt6 (**Fig. EV4A-B**), with a smaller effect on Pol2 in the downstream promoter. When examining Pol2 pausing under control conditions, tandem downstream genes had lower Pol2 pausing index than promoter controls. Spt6 degradation mildly reduced pausing at both tandem genes and their controls (see Methods and **Fig. EV4E-G**). Interestingly, no effect was seen for Rtf1 depletion (**Fig. EV4C-D**).*

We also revised figure S6 (now EV4) and use the same y-axis scale for all the plots:

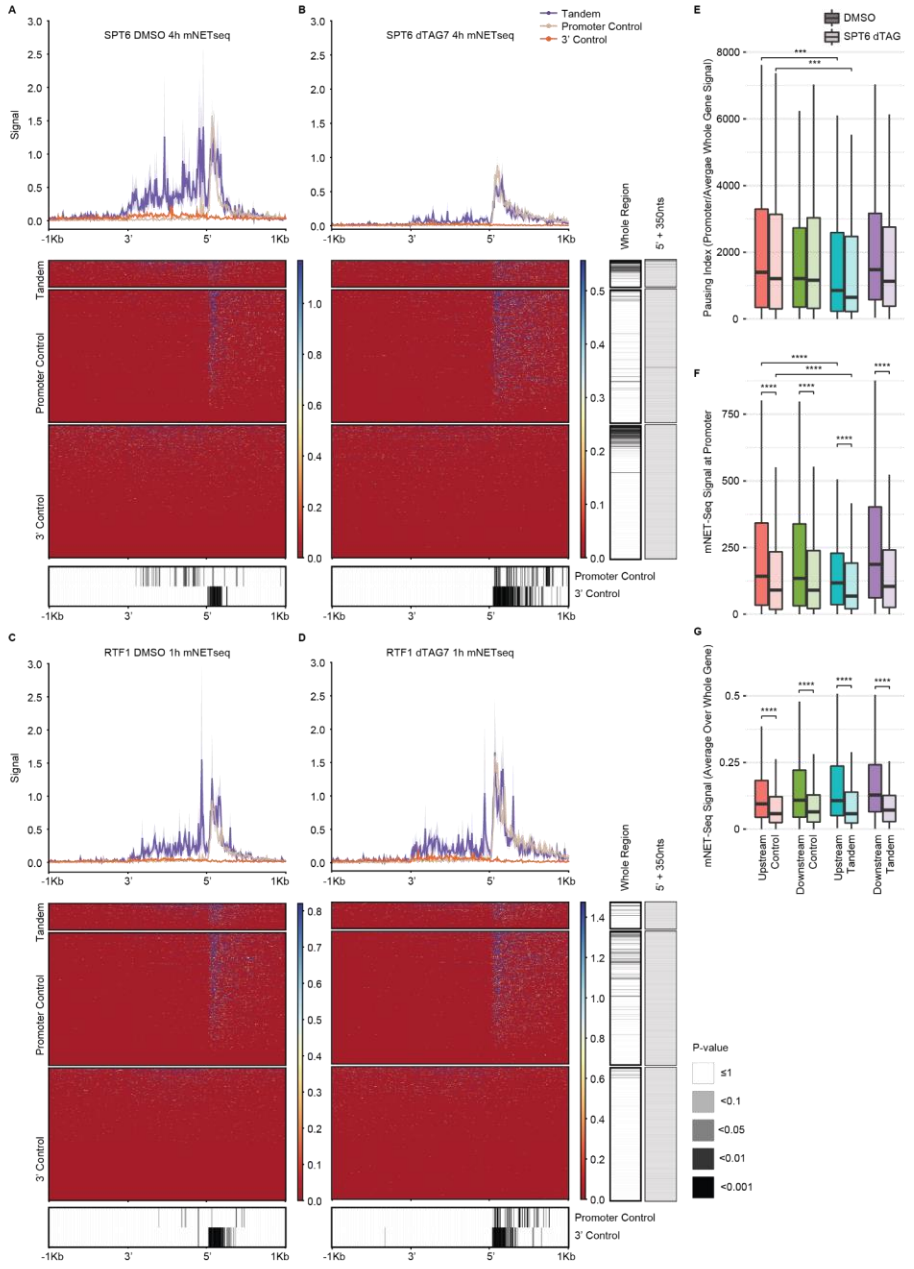

Figure EV4. SPT6 degradation is associated with Pol2 paucity at STIRs.
(A) Metagene analysis (top) and corresponding heatmap (center) showing mNET-seq median signal following control (DMSO) treatment in K562 co-expressed STIRs and flanking regions and in their controls. Bottom heatmap shows the corrected P-value of binned paired Wilcoxon rank-sum test. Data from (Žumer et al, 2021), using the CMA601 anti-CTD antibody as in Figs. 5 and S2.
(B) As in (A), for K562 cells 4h after treatment with SPT6 dTAG7. Centered heatmap is ordered by the gene order in the heatmap

of (A). Right heatmap is the corrected paired Wilcoxon rank-sum test per gene before and after treatment. Proportion of significantly changed signal for genes following the KD treatment was tested between the group of tandem genes and each control group and was significant in both cases (P=4.4×10-31 and P=1.1×10-9 for the promoter or the 3' control, respectively).
(C) As in (A), controls for the RTF1 1h treatment with DMSO.
(D) As in (C), 1h following RTF1 dTAG7 treatment. Proportion of significantly changed signal for genes following the KD treatment was tested between the group of tandem genes and each control group and had P=0.57 and P=1.6×10-4 for the promoter or the 3' control, respectively.
(E) Boxplots showing the Pol2 pausing index over upstream- (turquoise) or downstream- (purple) tandem genes or over their controls (red and purple, respectively), for either DMSO- (dark), or SPT6 KD- (light) treatment. Shown are corrected P-values obtained using Wilcoxon rank-sum test.
(F-G) mNET-seq signal over promoter regions (F) or mean signal across whole gene (G) (colors are as in (E)).

- Fig. 6. There is an impressive amount of work in this figure. The n needs to be clear from the legend. Are analyses based on RNA-seq data? If so, the authors are not in the position to distinguish if signal over the downstream gene corresponds to mRNA initiating from the 5´-SIRT, or may correspond to read-through transcription from the 3´-part of the SIRT configuration. For the selected set there appears to be an upregulation of the downstream gene upon NELF KD, which may be a nice finding to motivate further investigation. Perhaps data from the NELF-AID system (PMID: 32155413) could be used to support this conclusion with an orthogonal approach to deplete NELF that is arguably better suited to reveal the direct effects.

We now indicate in the legend of Figure 6A that the data are poly(A)-selected RNA-seq, and indicate the n (188 genes). We also now indicate in the text that the data are only informative for mature transcripts (*"These data from RNA-seq of poly(A)-selected transcripts, and so informative only for expression of mature RNA products."*). More broadly, beyond NELF-E, unfortunately such large-scale datasets on the effects of perturbations on nascent RNA are scarce. We also analyzed the suggested NELF-AID system data. Unfortunately, this study does not include RNA-seq data that could be directly compared to the ENCODE data that we use here, and the PRO-seq signal in gene bodies was too sparse to allow robust comparison or quantification on the level of individual genes, but we could see that NELF perturbations affected Pol2 occupancy in STIRs. We added this new analysis to the text:

*We next examined data from DLD-1 cells where NELF-C or NELF-E were tagged with an AID domain enabling inducible degradation (Aoi et al, 2020), and chromatin occupancy of various factors was examined before and after degradation (**Fig. S4B-E**). Occupancy of NELF-C and NELF-E was similar between STIRs and control promoters, with slightly lower occupancy in STIRs for (**Fig. S4B-C**). Pol2 with both S2P and S5P modifications was strongly enriched in STIRs compared to controls,*

*fitting the observations from the mNET-seq data (**Fig. S4D-E**) and S2P-modified Pol2 signal in STIRs was substantially more sensitive to loss of NELF-C than control promoters. We conclude that reduction in NELF complex levels leads to reduction of S2P-modified Pol2 which accumulates at STIRs and to increase in the expression of the downstream gene. Future studies may elucidate the mechanistic connection between these changes.*

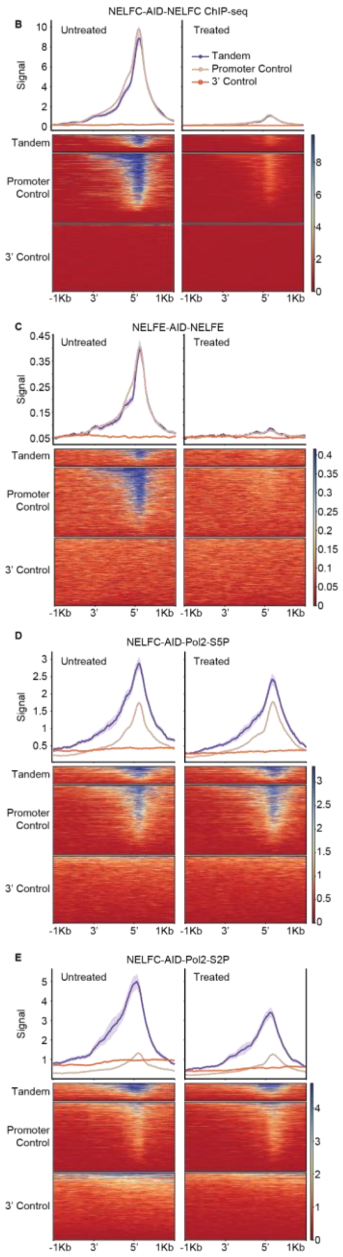

Figure S4. KD candidates for the transcription regulation of downstream tandem genes.

(B) Metagene analysis (top) and corresponding binding heatmap (bottom) of NELFC median ChIP-seq signal for NELFC AID-treated (right), or non-treated (left) data at STIRs between genes co-expressed in Dld1 cells and flanking 5' and 3' regions and at the control regions.

(C) As in (B), but for NELF-E.

(D-E) As in (B), but ChIP-seq was done for Pol2 S5P (D) and S2P (E) modification following NELF-C KD.

- Is there any experimental data to justify statements on Pol2 recycling in this manuscript? Perhaps some data (PMID: 30150253) aiming to resolve the dynamics of Pol2 transcription could be used to inform if upstream transcription facilitates downstream gene expression by Pol2 recycling. However, addressing this thoroughly will likely require substantial wet lab experiments.

We now analyzed the proposed dataset of Pol2 recycling. While these Pol2 occupancy data cannot be directly used to test if the same Pol2 that transcribed the upstream gene can be "reused" to transcribe the downstream one, they did allow us to test if the Pol2 accumulating at STIRs and at downstream promoters is found in a pre-initiation/poised state (which is susceptible to removal by high salt) or in an elongating state, we found that Pol2 in STIRs or at downstream tandem promoters was preferentially resistant to treatment with NaCl or glucose, suggesting that at least part of it is in an elongating state. We added these new results to the text:

*In order to study further the nature of the Pol2 accumulating at STIRs and at the downstream promoter, we analyzed data on Pol2 occupancy in HCT116 cells treated with conditions resulting in a high salt concentration (Erickson et al, 2018). Pol2 in STIRs and at the downstream promoter in a tandem pair was more resistant to NaCl or glucose treatments, that increase salt concentrations within the cells, than Pol2 pausing at the control promoters (**Fig. S3**). These results suggest that at least some of the Pol2 complexes in the aforementioned regions are in an "elongation" rather than in a "pre-initiation/poised" state, as the latter state is more sensitive to high salt concentrations (Erickson et al, 2018).*

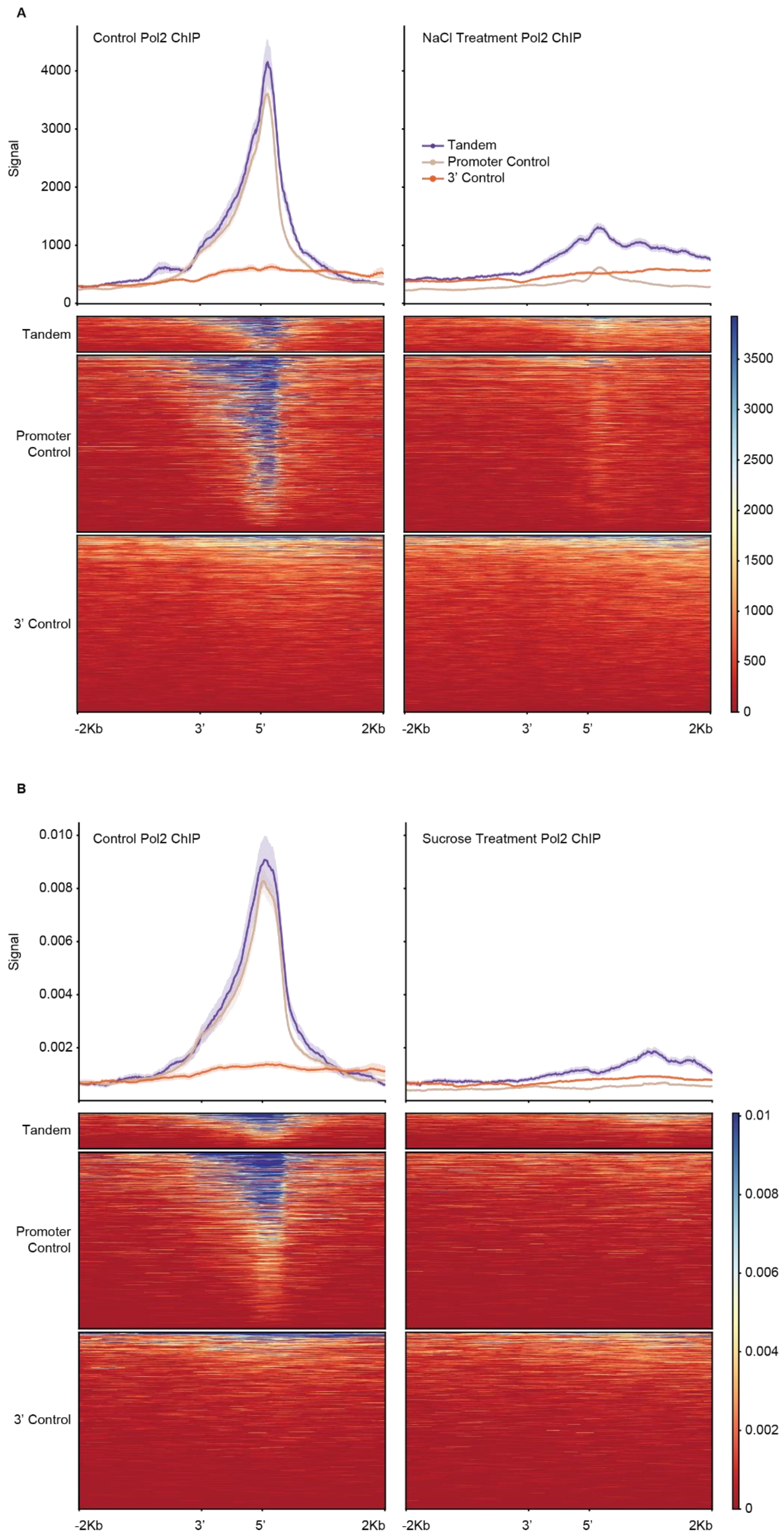

Figure S3. Evidence for elongating Pol2 at STIRs. (A) Profile (top) and metagene plot (bottom) for Pol2 ChIP for control treated (left) or NaCl treated (right) HCT116 cells (using Rabbit anti-pan CTD antibody; (Erickson et al. 2018), (Schroeder et al. 2000)). Plotted are the STIRs (purple) or the control promoters (beige) or control CPAs (orange) and flanking 2kb within the gene bodies.
(B) As in (A), but for Sucrose treatment.

Reviewer #3

Nissani and Ulitsky present a bioinformatics analysis of closely spaced tandemly oriented genes in the human genome. Such genes are potentially in position where they might be sensitized to transcriptional interference from terminating Pol2 from the upstream gene on initiation at the downstream gene. Analyses suggest that features of intergenic regions, while highly enriched for promoter attributes, are still distinguishable from control promoter sets. Much of the discussion, which is fine for speculation, touches on concept of Pol2 "recycling" to the point that it is prominently featured in the abstract ("Overall, we find that features associated with potential Pol2 recycling rather than those associated with avoidance of transcriptional interference are the predominant driving force shaping these regions"). The arguments supporting this conclusion are somewhat hard to understand and do not appear rise to the level of a conclusion stated in the abstract. It is not clear if there is any positive data to support recycling is what critical feature of intergenic regions vs potential buffering from interference close to promoter. The identification of modified Pol2 accumulating with promoters though diminishing with distance downstream from upstream gene suggests that promoters could have features specifically relevant to slowing or pausing transcription that might otherwise read through or disrupt promoter chromatin. This is a result that potentially deserves greater prominence. Comments for the authors on potential improvements to presentation are indicated below.

1.Analysis for potential GC-related bias in ChIP or other signals. The STIRs are enriched in GC similar to, but higher than, control promoters. If examining the ChIP enrichment for factors in STIRs over control, every single factor is higher in STIRs even if marginally. This potentially suggests there is a technical bias in STIR regions that slightly enriches for binding. Potentially, for ChIP minimally, or elsewhere where it can be examined, this can be assessed or normalized. For ChIP if using the median ratio of all factors for STIRs/controls this would potentially at least adjust for the apparent bias. It might also be important to consider if there is any way, do to increased variance or bias in control regions, or potential issues in the expression matching, if there were bias in mRNA stabilities between STIR and control sets, then expression matched regions might not necessarily be expected to be matched for chromatin binding if factor binding is more expression of synthesis rate than steady state RNA levels.

We have analyzed and now present data on nascent chromatin-associated RNA in the tandem pair and in control genes in Figure EV1C, which show that nascent RNA production rates in the tandem pairs are similar to the controls. As described in response to the comments of the other reviewers above, we also revised the

presentation of the ChIP-seq results, showing that while most profiled factors are biased to bind to promoters, the small subset that we highlight is enriched in STIRs.

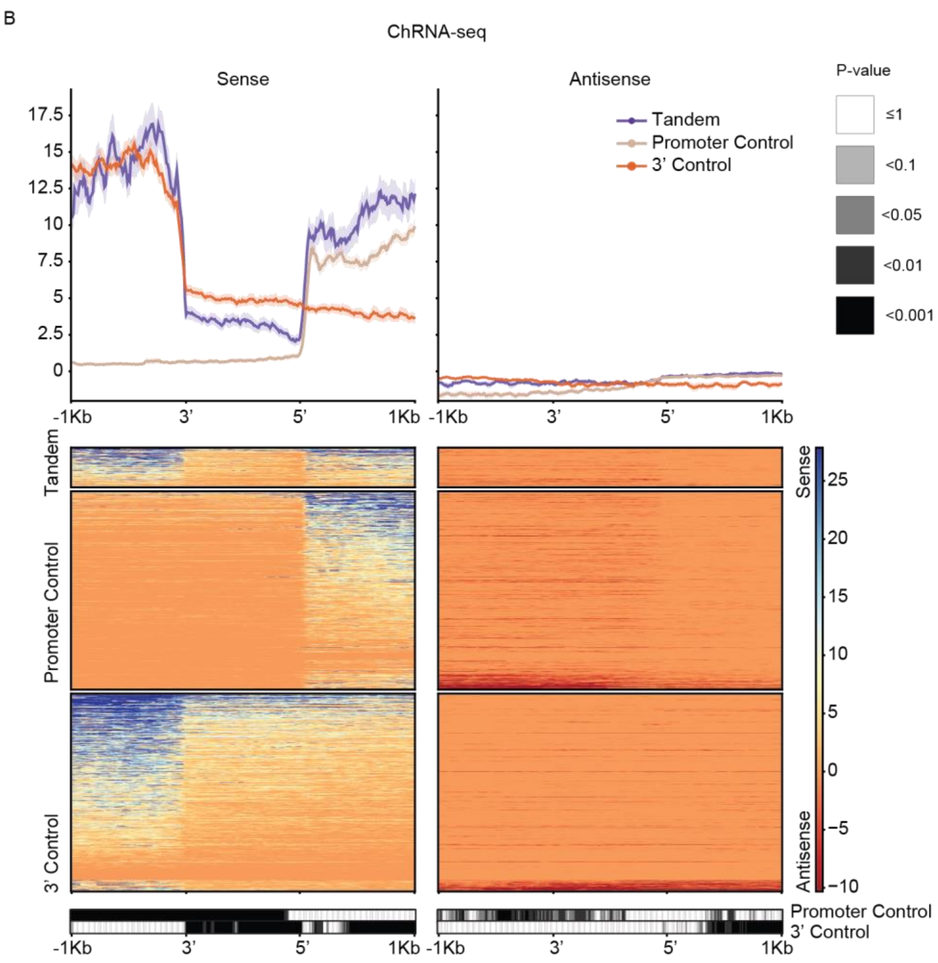

Figure EV1C Sense and antisense metagene profile (top) and heatmap (center) of median and standard error chromatin RNA-seq signal based on data from (Schlackow et al. 2017). Profile of the STIRs in HeLa cell line is plotted in between the CPA site of the upstream gene (5') to the TSS of the downstream tandem gene (3') and in 1kb flanking sequence. For the control genes, the TSS of promoter control genes are aligned to the TSS of the downstream gene (beige), and the CPA sites of 3' control genes are aligned to the CPA sites of the upstream gene (orange). Negative values indicate transcription on the antisense strand. Bottom plot shows the binned Bonferroni-corrected paired Wilcoxon rank-sum test P-value heatmap.

2. For motifs, is there an analysis that can ask if the marginal enrichment for motifs in STIRs over promoters is predicted solely by the increased GC content?

To address this question, we now use FIMO to compare the enrichment of the motifs identified by STREME between the sequences of STIRs and their controls, and shuffled, di-nucleotide preserving sequestions. We find that the motifs we highlight are substantially more common in STIRs that in the shuffled controls, and so they do not merely reflect sequence composition:

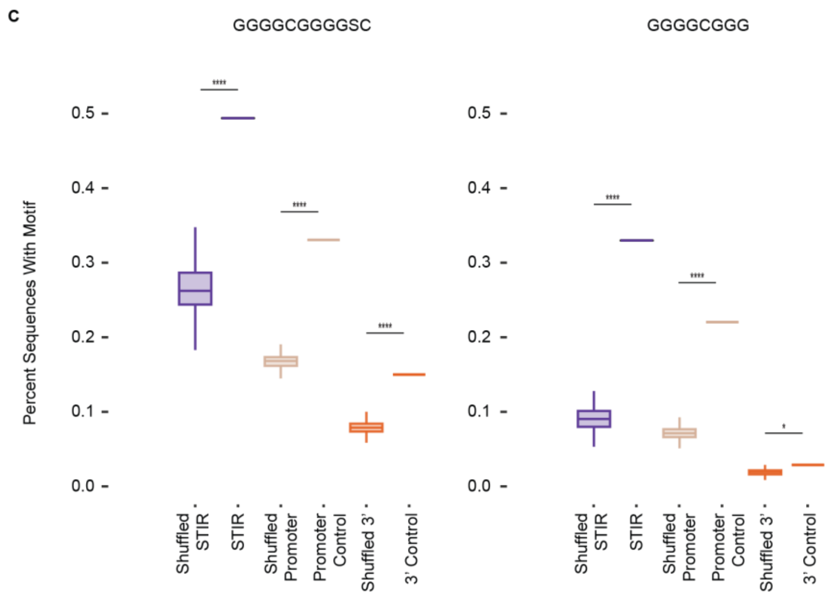

Fig. S1C Boxplots showing the fraction of dinucleotide-preserving shuffled sequences of STIRs or controls that contain either of the G-rich motifs. Bars correspond to the prevalence of either motif in the actual sequences. Shown also is the calculated P-value between occurrence prevalence at each group and its shuffled sequences.

3. In some cases figures can be confusing or potentially made more accessible to the reader.

We have improved numerous figures, including based on the feedback from the other reviewers.

4. There are many areas where a more broadly cast net for citation of literature could provide a cleaner context for the reader.

We added additional references, including based on the feedback of the other reviewers.

Other comments
Introduction
5. There are currently no citations for the allosteric model of termination. Please consider work of Martinson, H and colleagues.

We added the suggested citation.

6. There are studies that suggest in some cases interference might be a regulated phenomenon (work in yeast from Mellor et al (https://pubmed.ncbi.nlm.nih.gov/25407679/) or other cases where it appears that interference has evolved to function as part of gene regulation (Shuman 2020 is cited,

but this could be put into context). There are other data (two examples from Arabidopsis https://www.embopress.org/doi/full/10.15252/embr.201949315, https://www.nature.com/articles/s41467-019-12328-w) where it appears that evolution has employed systems to buffer downstream genes from interference that is apparent in mutants. These works and potentially others might benefit the reader. Certainly, the Arabidopsis papers present a strong case that at least in some species, downstream genes might require special protection or be sensitized to interference.

We thank the reviewer for suggesting these studies, that are now mentioned in the text:

The precision of the termination process is thought to have significant importance especially in the case of tandem genes, as it ensures the production of two stable transcripts, avoiding a readthrough transcript between a non-properly terminated upstream gene and continuing at the promoter of the downstream gene (Proudfoot, 2016; Shearwin et al, 2005). There are examples of such transcriptional interference between adjacent genes having regulatory roles in model organisms (Nguyen et al, 2014), and specific proteins as well as appropriate Pol2 speed were shown to ensure efficient transcriptional termination (Yu et al, 2019; Leng et al, 2020; Krzyszton et al, 2018), suggesting that uncontrolled termination can cause substantial crosstalk between adjacent transcription units.

7. p2, middle "The torpedo model connects between nascent RNA.....effect on transcriptional termination (Kim et al. 2004; West, Gromak, and Proudfoot 2004)" There may be additional papers that describe effects of termination defects on downstream genes, for example, knockdown of XRN3 causes altered expression of some genes and the accumulation of read-through transcripts (Krzyszton, M., Zakrzewska-Placzek, M., Kwasnik, A., Dojer, N., Karlowski, W. and Kufel, J. (2018), Defective XRN3-mediated transcription termination in Arabidopsis affects the expression of protein-coding genes. Plant J, 93: 1017-1031. https://doi.org/10.1111/tpj.13826).
We now mention this study in the paragraph discussed above.

Results
8. p4, middle "closely spaced (<2kb) genes (with the constraints imposed above).. We" Typo (extra period).
Fixed.

9. Figure 1. Is there any expression level bias between upstream and downstream tandem gene pair, either by mNET-seq or RNA-seq, beyond just asking if they are coexpressed or not, or if there is bias in upstream or downstream when one is expressed and the other is not?

We now performed this analysis. We find that there is no bias for a higher expression of the upstream or the downstream gene, while, as expected there is some correlation between their expression levels (page 5):

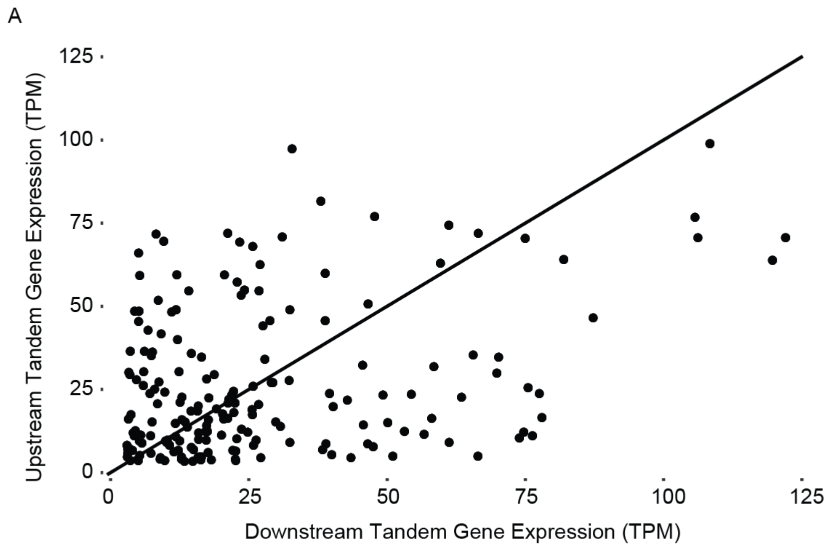

Figure EV1A: (A) Scatter plot showing the correlation between the TPM expression of downstream and upstream tandem gene pairs in HepG2 cell line (based on ENCODE data). Calculated Spearman correlation R=0.24 with Bonferroni corrected P-value of 0.007. Black line represents an R=1 correlation.

*As expected there was a mild yet significant correlation between the expression levels of the two tandem genes (**Fig. EV1A**, Spearman R=0.24 P=0.007, ENCODE HepG2 RNA-seq data), but there was no preference for the upstream or downstream transcripts to be more abundant (P=0.49 for Wilcoxon paired test comparing expression of the upstream and the downstream genes in HepG2 cells, Bonferroni-adjusted P>0.1 for each of seven other cell lines examined).*

10. Figure 3C. Because of the multiple types of controls used, it can be confusing when terms such as "Minimal STIR-to-Control ratio" is used. What is meant here? Based on potential GC bias and the dominant promoter nature of STIRs, potentially the control here should be the promoter control.

As mentioned above, we have now thoroughly revised the presentation of the ChIP-seq results in Figure 3 to address this and other comments.

11. p14, bottom "resulting in an overall 6.8 and 8.4-fold enrichment of Pol2 occupancy across the STIR, for the 3' control and promoter control, respectively (see Methods)"

From Figure 5 A and Figure 5S A, it looks like the Pol2 occupancy signal is closer to promoter control. Why does it have more (8.4-fold) enrichment compared with promoter control, but fewer (6.4-fold) enrichment compared with 3' control?

The sentence refers to the enrichment across the STIR region, that does not include the region downstream of the TSS of the downstream gene, where promoter control is indeed similar to the downstream gene in the tandem pair:

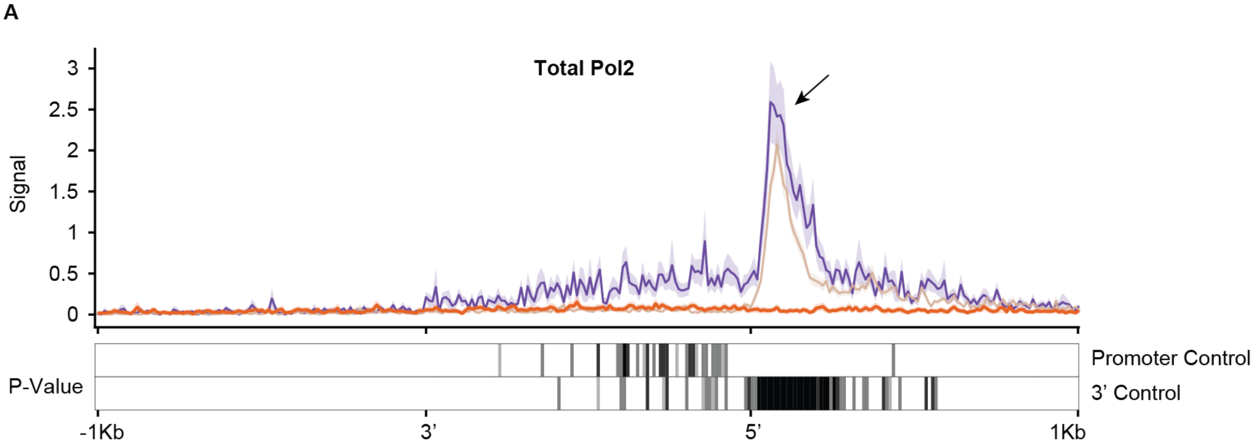

Figure 5A: Metagene analysis (top) showing the median Pol2 carboxy-terminal domain (phosphorylated and non-phosphorylated states) occupancy signal at the sense strand of STIRs or controls and their flanking regions. Bottom are the respective corrected paired Wilcoxon rank-sum test P-values. Data from Schlackow *et al.*, 2017.

12. p17, middle "with little to no effect on Pol2 in the downstream promoter". It looks like Pol2 at the downstream promoter is also reduced, the signal is above one before SPT6 degradation then reduced to about 0.6. This is at least not "no effect". What about within the gene or traveling ratio, or expression? Interestingly, spt6 mutants in yeast have reduced transcription interference in the Proudfoot system referenced (https://www.sciencedirect.com/science/article/pii/S0021925820764586) but an Spt6-degron paper from Wolf lab (not sure if cited here, 10.1016/j.molcel.2021.06.016) suggests situation could be more complicated in that Spt6 might also increase readthrough at genes.)

We agree with the reviewer that the promoter region of the downstream gene is also affected, and now updated Figure S6A (now Figure EV4) to show the same y axis scale for both control and perturbed cells. We also adjusted the text to fit the data. We also examined the traveling ratio in these data and found that Spt6 knockdown led to a slightly reduced pausing at both tandem and control genes, with an overall much smaller effect size than the reductions seen in occupancy at the promoters and in the gene bodies. Interestingly, downstream tandem genes have lower pausing index in control conditions, which we now note in the text:

When examining Pol2 pausing under control conditions, tandem downstream genes had lower Pol2 pausing index than promoter controls. Spt6 degradation mildly reduced pausing at both tandem genes and their controls (see Methods and ***Fig. EV4E-G****)*

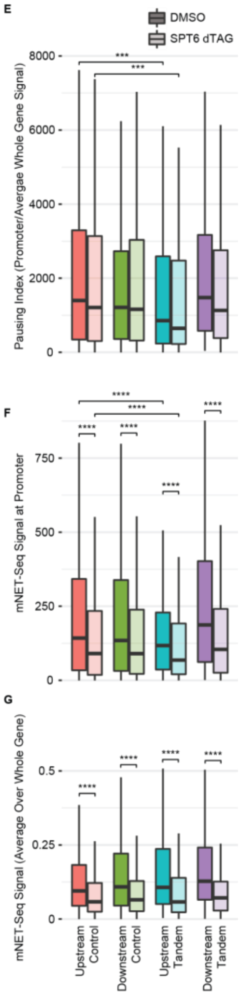

Figure EV4. SPT6 degradation is associated with Pol2 paucity at STIRs.

(E) Boxplots showing the Pol2 pausing index over upstream- (turquoise) or downstream- (purple) tandem genes or over their controls (red and purple, respectively), for either DMSO- (dark), or SPT6 KD- (light) treatment. Shown are corrected P-values obtained using Wilcoxon rank-sum test.

(F-G) mNET-seq signal over promoter regions (F) or mean signal across whole gene (G) (colors are as in (E)).

Discussion

13. p19, bottom and p20, top "Our observation of a slightly...Pol2 pausing at termination sites (Fig. 2D)." Please check figure callouts- for example, the metagene analysis of MAZ is in figure 2E but it is labeled as figure 2F in this paragraph of discussion.

Fixed.

14. p21, top "G-rich sequences that are also predominantly found near the downstream promoter (Fig. 2E)." This appears to mean 2D, not 2E.

Correct, Fixed.

Figures

15. Figure 2E. Potentially the label of tandem heatmap of metagene analysis could be moved upwards to make it clear given that the label overlaps with the promoter control heat map. Potentially label could be horizontal for this heatmap.

Fixed.

16. In some cases it is not apparent why P values are different over individual control regions where it seems that STIR signal is very similarly higher than either control region but one shows significance and another doesn't. This is sort of hard to intuitively understand.

The pairwise comparisons with the controls are based on all the data, whereas the plots are showing the median value. While this is indeed not always intuitive we prefer to show both values, as it highlights the regions where there is both a significant and substantial difference.

During our pre-decision cross-commenting process, Reviewers #2 and #3 made additional comments, which I have included below.

Reviewer #3

1) It is true that many in field use "TTS" to represent polyA site. The labeling should change to indicate that. However, since termination that might result in interference will be obligately downstream of the polyA site, it should be fine to use polyA site as proxy for measuring intergenic distances in this study.

We now accordingly use “CPA site” instead of the ambiguous “TTS” throughout the manuscript.

2) If nascent read through in intergenic regions is difficult to discern, authors might provide caveats to the observation that modified Pol2 is found near downstream promoters. The result for S2P and T4P is potentially striking enough that even with caveat that it is difficult to tell where it originated from, it should be reported. An indirect argument would be that this signal appears to diminish with distance from upstream gene, suggesting a possible connection.

We now provide evidence based on the nano-COP data that nascent readthrough between the tandem genes is indeed rare.

3) I have less of a concern about potential lncRNA or other upstream transcription confounding controls, only because this would cause underestimation of the enrichments they observe, not over estimation, but it is a good point to be careful about.

We agree with the reviewer and now mention this in the text:

> *In some cases, there could also be additional transcripts on the sense strand that we are not considering, which can affect the boundaries of the STIRs and potentially invalidate some of our control genes, but inspection of ChRNA-seq coverage suggests that such transcripts are either rare or very lowly expressed (**Fig. EV1C**).*

4) The authors might additionally consider asking if downstream genes of STIR pairs have greater or lesser bidirectional transcription- an additional difference could be that they are evolved to have enhanced directionality on the downstream side to diminish divergent transcription that could be antisense to the upstream gene. Additionally, it might be useful to show mNET seq data for the bottom strand for all the different Pol2 modifications, this should allow a more clear view of what is going on here.

We now show the ChRNA-seq data for both strands in Figure EV1C, and see that while the levels of antisense transcription are overall low in all gene groups, and are even lower in the downstream promoter in a tandem gene pair than for control promoters. We now mention this in the text. The mNET signals for the antisense/negative strand for the different modifications are already shown in Figure S5 (Now S2), and they are generally negligibly low compared to the sense strand.

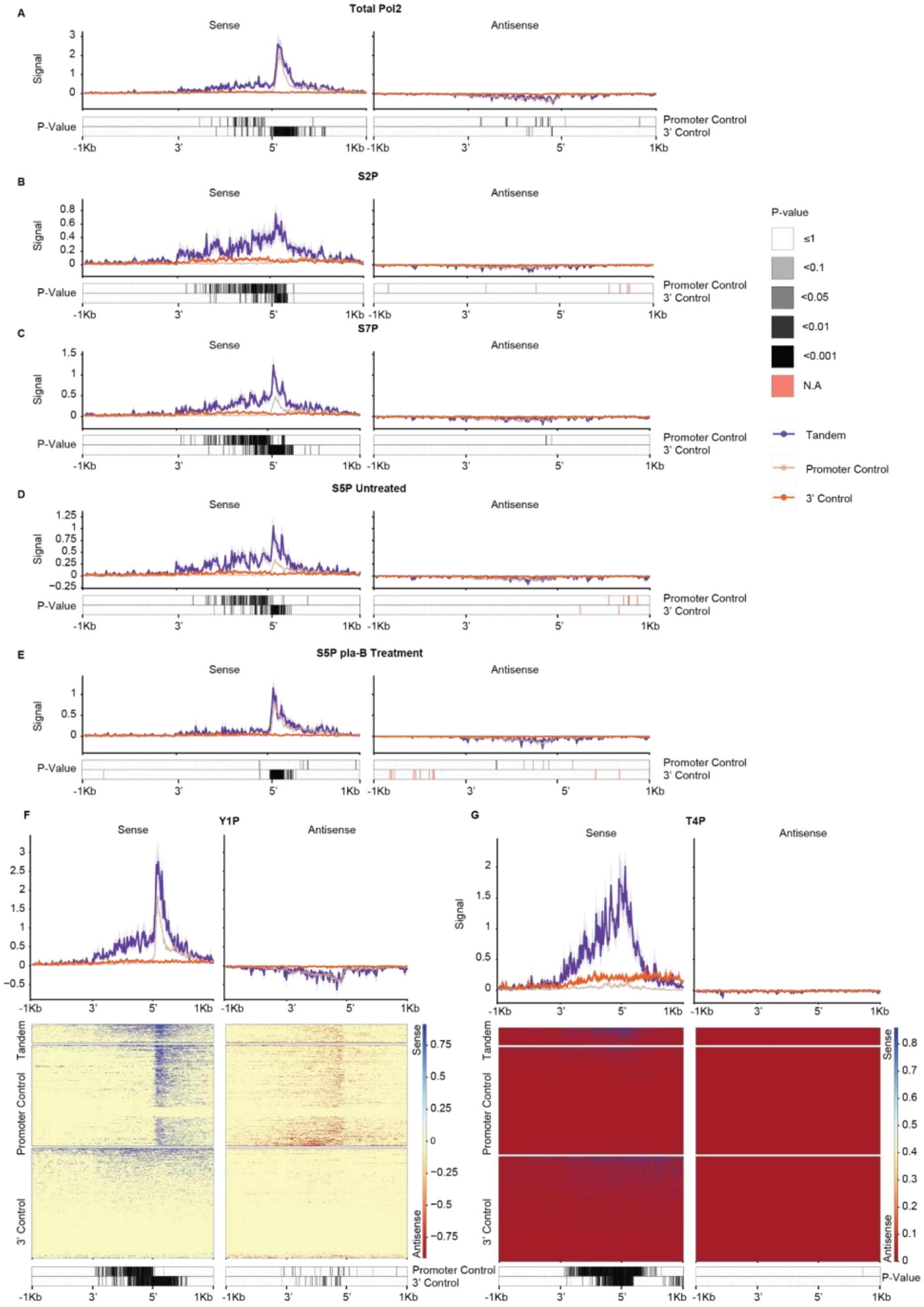

Figure S2: (A-E) Metagene analysis (top) showing the different median Pol2 carboxy-terminal domain modifications (phosphorylated and non-phosphorylated, S2P, S7P, S5P and S5P+Pla-B, respectively) occupancy signal and standard error at the sense and antisense strand of STIRs or controls and their flanking regions. Bottom are the respective corrected paired Wilcoxon rank-sum test P-values. NA P-values are marked in cases where data is missing. Data from Schlackow *et al.*, 2017.
(F-G) Metagene analysis (top) and corresponding heatmap (center) of median Pol2 CTD modifications (Y1P and T4P, respectively) mNET-seq signal and standard error at the sense and antisense strands of STIRs and controls, and their flanking regions. Negative values indicate transcription on the antisense strand. Bottom are the respective corrected paired Wilcoxon rank-sum test P-values. Data from Schlackow *et al.*, 2017.

Reviewer #2

A comment on the cross-comment points 2) and 3). The CTD signal mentioned in 2) could for instance stem from the missed transcription units mentioned in point 3). A correct segmentation with the data and know-how of 2021 will be necessary.

As mentioned in the comments above, we agree that a segmentation would be theoretically beneficial, but because of the predominantly short-read data that is available and the quantitative nature of transcription, it is exceptionally difficult to segment transcripts in gene-dense regions, and therefore we argue that it is out of scope of the current study. Based on the chRNA-seq data and nano-COP analysis we now show, transcription units joining the two genes together appear to be very rare.

2nd Mar 2022

Manuscript Number: MSB-2021-10682R
Title: Unique features of transcription termination and initiation at closely spaced tandem human genes
Author: Noa Nissani
Igor Ulitsky

Thank you for sending us your revised manuscript. We have now heard back from the two reviewers who agreed to evaluate your study. As you will see below, the reviewers are overall satisfied with the revisions made and think that the study is now suitable for publication.

Before we can formally accept your manuscript, we would ask you to address the following issues:
1. The remaining concerns of Reviewer#3, especially regarding their comment on "Pol II recycling" in the abstract.

On a more editorial level:

Click on the link below to submit your revised paper.

https://msb.msubmit.net/cgi-bin/main.plex

As a matter of course, please make sure that you have correctly followed the instructions for authors as given on the submission website.

Thank you for submitting this paper to Molecular Systems Biology. I look forward to receiving the revised version soon.

Kind regards,
Jingyi

Jingyi Hou
Editor
Molecular Systems Biology

If you do choose to resubmit, please click on the link below to submit the revision online before 1st Apr 2022.

https://msb.msubmit.net/cgi-bin/main.plex

IMPORTANT: When you send your revision, we will require the following items:
1. the manuscript text in LaTeX, RTF or MS Word format
2. a letter with a detailed description of the changes made in response to the referees. Please specify clearly the exact places in the text (pages and paragraphs) where each change has been made in response to each specific comment given
3. three to four 'bullet points' highlighting the main findings of your study
4. a short 'blurb' text summarizing in two sentences the study (max. 250 characters)
5. a 'thumbnail image' (550px width and max 400px height, Illustrator, PowerPoint or jpeg format), which can be used as 'visual title' for the synopsis section of your paper.
6. Please include an author contributions statement after the Acknowledgements section (see https://www.embopress.org/page/journal/17444292/authorguide#manuscriptpreparation)
7. Please complete the CHECKLIST available at (https://bit.ly/EMBOPressAuthorChecklist).
Please note that the Author Checklist will be published alongside the paper as part of the transparent process (https://www.embopress.org/page/journal/17444292/authorguide#transparentprocess).
8. When assembling figures, please refer to our figure preparation guideline in order to ensure proper formatting and readability in print as well as on screen:
https://bit.ly/EMBOPressFigurePreparationGuideline
See also figure legend guidelines: https://www.embopress.org/page/journal/17444292/authorguide#figureformat
9. Please note that corresponding authors are required to supply an ORCID ID for their name upon submission of a revised manuscript (EMBO Press signed a joint statement to encourage ORCID adoption).
(https://www.embopress.org/page/journal/17444292/authorguide#editorialprocess)
Currently, our records indicate that the ORCID for your account is 0000-0003-0555-6561.

Please click the link below to modify this ORCID:
Link Not Available

The system will prompt you to fill in your funding and payment information. This will allow Wiley to send you a quote for the article processing charge (APC) in case of acceptance. This quote takes into account any reduction or fee waivers that you may be eligible for. Authors do not need to pay any fees before their manuscript is accepted and transferred to the publisher.

As a matter of course, please make sure that you have correctly followed the instructions for authors as given on the submission website.

*** PLEASE NOTE *** As part of the EMBO Press transparent editorial process initiative (see our Editorial at

https://dx.doi.org/10.1038/msb.2010.72 , Molecular Systems Biology will publish online a Review Process File to accompany accepted manuscripts. When preparing your letter of response, please be aware that in the event of acceptance, your cover letter/point-by-point document will be included as part of this File, which will be available to the scientific community. More information about this initiative is available in our Instructions to Authors. If you have any questions about this initiative, please contact the editorial office (msb@embo.org).

Reviewer #1:

The authors have thoroughly addressed my comments. All in all, the additional data panels along with the clarifications added to the main text have improved the manuscript.

Reviewer #3:

Abstract really should remove concept of recycling. Inclusion in discussion is fine. Revision was done in good faith and extensively.

1. p10 Middle. "Notably, ENCODE ChIP-seq data of MAZ in HepG2 cells displayed a higher binding signal in STIRs compared to both types of controls ..... Sp1 also showed a slightly higher binding signal at the promoter regions..... (Fig. 2E). "

MAZ and SP1 have higher binding signals in STIRs compared to 3' control, and just slightly higher than promoter control but it is not clear if significant based on the p-values in the figure.

2. p12 Top. " ... including AGO2, AGO1, RBM22, BCL3, MYNN, and NFATC1 (Fig. 3A-G and EV2C-FC-G)."

The figure callout appears to include both old and new due to missed track changes acceptance.

3. p21 Middle. "Occupancy of NELF-C and NELF-E was similar between STIRs and control promoters, with slightly lower occupancy in STIRs for [missing word]

Sentence is incomplete

Editorial comments:

1. The remaining concerns of Reviewer#3, especially regarding their comment on "Pol II recycling" in the abstract.
Fixed

On a more editorial level:
2. please provide up to 5 keywords and incorporate them in the main text.
Added

3. Authors' contribution: the contribution of every author must be detailed in a separate section under the heading "Author Contributions".
Added

4. We updated our journal's competing interests policy in January 2022 and request authors to consider both actual and perceived competing interests. Please review the policy https://www.embopress.org/competing-interests and add your competing interests if necessary.

Please add a disclosure statement using the heading "Disclosure statement and competing interests".
Added

5. Figures:
- Main figures and EV figures must be uploaded as single files.
- Remove the figures from the main manuscript file.
- Combine the figure legends and place them at the end of the manuscript file.
Adjusted accordingly

6. Remove "data not shown". As per our guidelines on "Unpublished Data", the journal does not permit citation of "data not shown". All data referred to in the paper should be displayed in the main or Expanded View figures.
Removed (the relevant data for the statements was already presented)

7. Supplementary figures:

- The supplementary figures (not EV figures) should be bundled together with their legends in a single PDF file called *Appendix*, starting with a short Table of Content. Each legend should be below the corresponding Figure in the Appendix. Appendix figures should be referred to in the main text as: "Appendix Figure S1, Appendix Figure S2," etc. See detailed instructions regarding expanded view here:
https://www.embopress.org/page/journal/17444292/authorguide#expandedview.
- Please remove the Figures S1-7 and their legends from the main manuscript file.
Done.

8. EV tables:

- Since they are quite large, please update them to "Dataset EV #". Please update the callouts in the manuscript text accordingly.
Done

- Please upload them individually and insert the legend into each corresponding dataset in a separate sheet labeled "legend".
Done

- Please use the nomenclature "Dataset EV#" in the legend sheet.
Done

9. Data availability

- Please add a formal "Data Availability" section (placed after Materials & Method).
- Since this study does not generate large-scale datasets, please only include the following sentence in this section- "This study includes no data deposited in external repositories".
Attached.

10. I have slightly modified the synopsis text (see attached). Please let me know if it is fine or if you would like to introduce further modifications.
Approved.

11. Our data editors have seen the manuscript, and they have made some comments and suggestions that need to be addressed (see attached file). Please send back a revised version (in track change mode), as we will need to go through the changes.
Done.

Reviewer #1:

The authors have thoroughly addressed my comments. All in all, the additional data panels along with the clarifications added to the main text have improved the manuscript.

Reviewer #3:

Abstract really should remove concept of recycling. Inclusion in discussion is fine. Revision was done in good faith and extensively.
We reworded the sentence and avoid the use of the term “recycling”

1. p10 Middle. "Notably, ENCODE ChIP-seq data of MAZ in HepG2 cells displayed a higher binding signal in STIRs compared to both types of controls ..... Sp1 also showed a slightly higher binding signal at the promoter regions..... (Fig. 2E). "

MAZ and SP1 have higher binding signals in STIRs compared to 3' control, and just slightly higher than promoter control but it is not clear if significant based on the p-values in the figure.

We reworded these sentences and specifically that the difference compared to promoter controls is not significant for both MAZ and Sp1.

2. p12 Top. " ... including AGO2, AGO1, RBM22, BCL3, MYNN, and NFATC1 (Fig. 3A-G and EV2C-FC-G)."

The figure callout appears to include both old and new due to missed track changes acceptance.
Fixed

3. p21 Middle. "Occupancy of NELF-C and NELF-E was similar between STIRs and control promoters, with slightly lower occupancy in STIRs for [missing word]
Fixed

10th Mar 2022
Manuscript number: MSB-2021-10682RR
Title: Unique features of transcription termination and initiation at closely spaced tandem human genes

Thank you again for sending us your revised manuscript. We are now satisfied with the modifications made and I am pleased to inform you that your paper has been accepted for publication.

*** PLEASE NOTE *** As part of the EMBO Publications transparent editorial process initiative (see our Editorial at https://dx.doi.org/10.1038/msb.2010.72), Molecular Systems Biology publishes online a Review Process File with each accepted manuscripts. This file will be published in conjunction with your paper and will include the anonymous referee reports, your point- by-point response and all pertinent correspondence relating to the manuscript. If you do NOT want this File to be published, please inform the editorial office at msb@embo.org within 14 days upon receipt of the present letter.

Should you be planning a Press Release on your article, please get in contact with msb@wiley.com as early as possible, in order to coordinate publication and release dates.

LICENSE AND PAYMENT:
All articles published in Molecular Systems Biology are fully open access: immediately and freely available to read, download and share.

Molecular Systems Biology charges an article processing charge (APC) to cover the publication costs. You, as the corresponding author for this manuscript, should have already received a quote with the article processing fee separately. Please let us know in case this quote has not been received.

Once your article is at Wiley for editorial production you will receive an email from Wiley's Author Services system, which will ask you to log in and will present you with the publication license form for completion. Within the same system the publication fee can be paid by credit card, an invoice or pro forma can be requested.

Payment of the publication charge and the signed Open Access Agreement form must be received before the article can be published online.

Molecular Systems Biology articles are published under the Creative Commons licence CC BY, which facilitates the sharing of scientific information by reducing legal barriers, while mandating attribution of the source in accordance to standard scholarly practice.

Proofs will be forwarded to you within the next 2-3 weeks.

Thank you very much for submitting your work to Molecular Systems Biology.

Kind regards,
Jingyi

Jingyi Hou
Editor
Molecular Systems Biology

EMBO Press Author Checklist

Corresponding Author Name: Igor Ulitsky	
Journal Submitted to: Molecular Systems Biology	
Manuscript Number: MSB-2021-10682	

USEFUL LINKS FOR COMPLETING THIS FORM
The EMBO Journal - Author Guidelines
EMBO Reports - Author Guidelines
Molecular Systems Biology - Author Guidelines
EMBO Molecular Medicine - Author Guidelines

Reporting Checklist for Life Science Articles (updated January 2022)
This checklist is adapted from Materials Design Analysis Reporting (MDAR) Checklist for Authors. MDAR establishes a minimum set of requirements in transparent reporting in the life sciences (see Statement of Task: 10.31222/osf.io/9sm4x). Please follow the journal's guidelines in preparing your manuscript.
Please note that a copy of this checklist will be published alongside your article.

Abridged guidelines for figures
1. Data
The data shown in figures should satisfy the following conditions:
- the data were obtained and processed according to the field's best practice and are presented to reflect the results of the experiments in an accurate and unbiased manner.
- ideally, figure panels should include only measurements that are directly comparable to each other and obtained with the same assay.
- plots include clearly labeled error bars for independent experiments and sample sizes. Unless justified, error bars should not be shown for technical replicates.
- if n<5, the individual data points from each experiment should be plotted. Any statistical test employed should be justified.
- Source Data should be included to report the data underlying figures according to the guidelines set out in the authorship guidelines on Data Presentation.

2. Captions
Each figure caption should contain the following information, for each panel where they are relevant:
- a specification of the experimental system investigated (eg cell line, species name).
- the assay(s) and method(s) used to carry out the reported observations and measurements.
- an explicit mention of the biological and chemical entity(ies) that are being measured.
- an explicit mention of the biological and chemical entity(ies) that are altered/varied/perturbed in a controlled manner.
- the exact sample size (n) for each experimental group/condition, given as a number, not a range;
- a description of the sample collection allowing the reader to understand whether the samples represent technical or biological replicates (including how many animals, litters, cultures, etc.).
- a statement of how many times the experiment shown was independently replicated in the laboratory.
- definitions of statistical methods and measures:
  - common tests, such as t-test (please specify whether paired vs. unpaired), simple χ2 tests, Wilcoxon and Mann-Whitney tests, can be unambiguously identified by name only, but more complex techniques should be described in the methods section;
  - are tests one-sided or two-sided?
  - are there adjustments for multiple comparisons?
  - exact statistical test results, e.g., P values = x but not P values < x;
  - definition of 'center values' as median or average;
  - definition of error bars as s.d. or s.e.m.

Please complete ALL of the questions below.
Select "Not Applicable" only when the requested information is not relevant for your study.

Materials

Newly Created Materials	Information included in the manuscript?	In which section is the information available? (Reagents and Tools Table, Materials and Methods, Figures, Data Availability Section)
New materials and reagents need to be available; do any restrictions apply?	Not Applicable	

Antibodies	Information included in the manuscript?	In which section is the information available? (Reagents and Tools Table, Materials and Methods, Figures, Data Availability Section)
For **antibodies** provide the following information:<br>- Commercial antibodies: RRID (if possible) or supplier name, catalogue number and or/clone number<br>- Non-commercial: RRID or citation	Not Applicable	

DNA and RNA sequences	Information included in the manuscript?	In which section is the information available? (Reagents and Tools Table, Materials and Methods, Figures, Data Availability Section)
**Short novel DNA or RNA including primers, probes**: provide the sequences.	Not Applicable	

Cell materials	Information included in the manuscript?	In which section is the information available? (Reagents and Tools Table, Materials and Methods, Figures, Data Availability Section)
**Cell lines:** Provide species information, strain. Provide accession number in repository **OR** supplier name, catalog number, clone number, and/**OR** RRID.	Not Applicable	
**Primary cultures:** Provide species, strain, sex of origin, genetic modification status.	Not Applicable	
Report if the cell lines were recently **authenticated** (e.g., by STR profiling) and tested for mycoplasma contamination.	Not Applicable	

Experimental animals	Information included in the manuscript?	In which section is the information available? (Reagents and Tools Table, Materials and Methods, Figures, Data Availability Section)
**Laboratory animals or Model organisms:** Provide species, strain, sex, age, genetic modification status. Provide accession number in repository **OR** supplier name, catalog number, clone number, **OR** RRID.	Not Applicable	
**Animal observed in or captured from the field:** Provide species, sex, and age where possible.	Not Applicable	
Please detail **housing and husbandry conditions**.	Not Applicable	

Plants and microbes	Information included in the manuscript?	In which section is the information available? (Reagents and Tools Table, Materials and Methods, Figures, Data Availability Section)
**Plants:** provide species and strain, ecotype and cultivar where relevant, unique accession number if available, and source (including location for collected wild specimens).	Not Applicable	
**Microbes:** provide species and strain, unique accession number if available, and source.	Not Applicable	

Human research participants	Information included in the manuscript?	In which section is the information available? (Reagents and Tools Table, Materials and Methods, Figures, Data Availability Section)
If collected and within the bounds of privacy constraints report on age, sex and gender or ethnicity for all study participants.	Not Applicable	

Core facilities	Information included in the manuscript?	In which section is the information available? (Reagents and Tools Table, Materials and Methods, Figures, Data Availability Section)
If your work benefited from core facilities, was their service mentioned in the acknowledgments section?	Not Applicable	

Design

Study protocol	Information included in the manuscript?	In which section is the information available? (Reagents and Tools Table, Materials and Methods, Figures, Data Availability Section)
If study protocol has been **pre-registered, provide DOI in the manuscript**. For clinical trials, provide the trial registration number **OR** cite DOI.	Not Applicable	
Report the **clinical trial registration number** (at ClinicalTrials.gov or equivalent), where applicable.	Not Applicable	

Laboratory protocol	Information included in the manuscript?	In which section is the information available? (Reagents and Tools Table, Materials and Methods, Figures, Data Availability Section)
Provide DOI OR other citation details if **external detailed step-by-step protocols** are available.	Not Applicable	

Experimental study design and statistics	Information included in the manuscript?	In which section is the information available? (Reagents and Tools Table, Materials and Methods, Figures, Data Availability Section)

Include a statement about **sample size** estimate even if no statistical methods were used.	Not Applicable	
Were any steps taken to minimize the effects of subjective bias when allocating animals/samples to treatment (e.g. **randomization procedure**)? If yes, have they been described?	Not Applicable	
Include a statement about **blinding** even if no blinding was done.	Not Applicable	
Describe **inclusion/exclusion criteria** if samples or animals were excluded from the analysis. Were the criteria pre-established?<br><br>If sample or data points were omitted from analysis, report if this was due to attrition or intentional exclusion and provide justification.	Not Applicable	
For every figure, are **statistical tests** justified as appropriate? Do the data meet the assumptions of the tests (e.g., normal distribution)? Describe any methods used to assess it. Is there an estimate of variation within each group of data? Is the variance similar between the groups that are being statistically compared?	Yes	Results, Figure legends

Sample definition and in-laboratory replication	Information included in the manuscript?	In which section is the information available? (Reagents and Tools Table, Materials and Methods, Figures, Data Availability Section)
In the figure legends: state number of times the experiment was **replicated** in laboratory.	Not Applicable	
In the figure legends: define whether data describe **technical or biological replicates**.	Yes	Results, Figure legends

Ethics

Ethics	Information included in the manuscript?	In which section is the information available? (Reagents and Tools Table, Materials and Methods, Figures, Data Availability Section)
Studies involving **human participants**: State details of **authority granting ethics approval** (IRB or equivalent committee(s), provide reference number for approval.	Not Applicable	
Studies involving **human participants**: Include a statement confirming that **informed consent** was obtained from all subjects and that the experiments conformed to the principles set out in the WMA Declaration of Helsinki and the Department of Health and Human Services Belmont Report.	Not Applicable	
Studies involving **human participants:** For publication of **patient photos**, include a statement confirming that consent to publish was obtained.	Not Applicable	
Studies involving experimental **animals**: State details of **authority granting ethics approval** (IRB or equivalent committee(s), provide reference number for approval. Include a statement of compliance with ethical regulations.	Not Applicable	
Studies involving **specimen and field samples:** State if relevant **permits** obtained, provide details of authority approving study; if none were required, explain why.	Not Applicable	

Dual Use Research of Concern (DURC)	Information included in the manuscript?	In which section is the information available? (Reagents and Tools Table, Materials and Methods, Figures, Data Availability Section)
Could your study fall under dual use research restrictions? Please check biosecurity documents and list of **select agents and toxins** (CDC): https://www.selectagents.gov/sat/list.htm	Not Applicable	
If you used a select agent, is the security level of the lab appropriate and reported in the manuscript?	Not Applicable	
If a study is subject to dual use research of concern regulations, is the name of the **authority granting approval and reference number** for the regulatory approval provided in the manuscript?	Not Applicable	

Reporting

The MDAR framework recommends adoption of discipline-specific guidelines, established and endorsed through community initiatives. Journals have their own policy about requiring specific guidelines and recommendations to complement MDAR.

Adherence to community standards	Information included in the manuscript?	In which section is the information available? (Reagents and Tools Table, Materials and Methods, Figures, Data Availability Section)
State if relevant guidelines or checklists (e.g., **ICMJE, MIBBI, ARRIVE, PRISMA**) have been followed or provided.	Not Applicable	
For **tumor marker prognostic studies**, we recommend that you follow the **REMARK** reporting guidelines (see link list at top right). See author guidelines, under 'Reporting Guidelines'. Please confirm you have followed these guidelines.	Not Applicable	
For **phase II and III randomized controlled trials**, please refer to the **CONSORT** flow diagram (see link list at top right) and submit the CONSORT checklist (see link list at top right) with your submission. See author guidelines, under 'Reporting Guidelines'. Please confirm you have submitted this list.	Not Applicable	

Data Availability

Data availability	Information included in the manuscript?	In which section is the information available? (Reagents and Tools Table, Materials and Methods, Figures, Data Availability Section)
Have **primary datasets** been deposited according to the journal's guidelines (see 'Data Deposition' section) and the respective accession numbers provided in the Data Availability Section?	Not Applicable	
Were **human clinical and genomic datasets** deposited in a public access-controlled repository in accordance to ethical obligations to the patients and to the applicable consent agreement?	Not Applicable	
Are **computational models** that are central and integral to a study available without restrictions in a machine-readable form? Were the relevant accession numbers or links provided?	Not Applicable	
If publicly available data were reused, provide the respective **data citations in the reference list**.	Not Applicable	
